# Fluorescent Bis-Calix[4]arene-Carbazole Conjugates: Synthesis and Inclusion Complexation Studies with Fullerenes C_60_ and C_70_

**DOI:** 10.3390/molecules26165000

**Published:** 2021-08-18

**Authors:** Patrícia D. Barata, Alexandra I. Costa, Sérgio Costa, José V. Prata

**Affiliations:** 1 Departamento de Engenharia Química, Instituto Superior de Engenharia de Lisboa, Instituto Politécnico de Lisboa, R. Conselheiro Emídio Navarro, 1, 1959-007 Lisboa, Portugal; pbarata@deq.isel.ipl.pt (P.D.B.); acosta@deq.isel.ipl.pt (A.I.C.); sethcosta@hotmail.com (S.C.); 2 Centro de Química-Vila Real, Universidade de Trás-os-Montes e Alto Douro, 5001-801 Vila Real, Portugal

**Keywords:** calix[4]arene, carbazole, fluorescence, fullerene, supramolecular, host–guest chemistry

## Abstract

Supramolecular chemistry has become a central theme in chemical and biological sciences over the last decades. Supramolecular structures are being increasingly used in biomedical applications, particularly in devices requiring specific stimuli-responsiveness. Fullerenes, and supramolecular assemblies thereof, have gained great visibility in biomedical sciences and engineering. Sensitive and selective methods are required for the study of their inclusion in complexes in various application fields. With this in mind, two new fluorescent bis-calix[4]arene-carbazole conjugates (**4** and **5**) have been designed. Herein, their synthesis and ability to behave as specific hosts for fullerenes C_60_ and C_70_ is described. The optical properties of the novel compounds and their complexes with C_60_ and C_70_ were thoroughly studied by UV-Vis and steady-state and time-resolved fluorescence spectroscopies. The association constants (*K*_a_) for the complexation of C_60_ and C_70_ by **4** and **5** were determined by fluorescence techniques. A higher stability was found for the C_70_@**4** supramolecule (*K*_a_ = 5.6 × 10^4^ M^−1^; Δ*G* = −6.48 kcal/mol). Evidence for the formation of true inclusion complexes between the host **4** and C_60_/C_70_ was obtained from NMR spectroscopy performed at low temperatures. The experimental findings were fully corroborated by density functional theory (DFT) models performed on the host–guest assemblies (C_60_@**4** and C_70_@**4**).

## 1. Introduction

Supramolecular chemistry represents one of the main topics of modern chemical sciences. Crossing traditional frontiers of organic, inorganic, and physical chemistry, supramolecular chemistry affords opportunities to create new supramolecules and nanostructures for applications as functional materials in chemistry, biology, and nanomedicine [1,2,3]. Reversible, usually weak, non-covalent host–guest interactions constitute the basis for the development of supramolecular architectures. The nature of such interactions ranges from electrostatic interactions, hydrogen bonding, cation-π, CH-π and π-π interactions, dispersion interactions (van der Waals interactions), and hydrophobic effects, resulting in a wide range of binding energies (around 0.5 kcal/mol for dispersion forces up to 70 kcal/mol for ion-ion interactions) [4].

Over the years, different types of macrocyclic scaffolds have been designed for serving as hosts in supramolecular events. Calixarenes [2,5,6,7,8,9], crown ethers [2,9], cyclodextrins [2,8,9], cyclophanes [2,9], cucurbit[n]urils [2,8,9] and pillar[n]arenes [2,9] are representative examples of such molecular machinery. Calix[n]arenes, cyclodextrins, and cucurbit[n]urils have, for example, been used as efficient hosts in biomedical applications, namely as drug delivering systems (e.g., drug carriers and drug bioavailability enhancers), bioimaging, and photodynamic therapy [8,10,11,12,13].

Calixarenes, in particular, having bowl-shaped cavities of various sizes, constitute one of the best known and recognized macrocyclic hosts, able to encapsulate a wide variety of guests (e.g., neutral, ionic molecular species, and ions) through the formation of inclusion complexes. Fluorescent calixarenes are particularly attractive for (bio)molecular sensing [6]. Highly sensitive and selective responses are expected by synergistic effects between the calixarene molecular receptor and the covalently linked fluorogenic unit, allowing high transduction signals to be retrieved upon binding to a particular guest. These types of molecular sensing systems have been recently used by us in the detection of explosives [14], pollutants [15], proteins [16], and metals [17].

Fullerenes, as a new class of carbon allotropes, emerged after the discovery of buckminsterfullerene-C_60_ by Kroto, Smalley, and Curl et al. in 1985 [18]. Since then, their chemistry has been explored in many directions and application fields. In biomedical science and engineering, fullerenes and their derivatives have had a tremendous impact in drug delivery, cancer photodynamic therapy, and biosensing [19,20]. The development of expedite methods for the study of their inclusion compounds with a variety of hosts for future applications in diverse areas is essential. Moreover, their sensitive and selective detection and tracking in various media, at micro/sub-micromolar to nanomolar scales, are still required. Indeed, although a great number of reports have appeared in the literature showing the formation of inclusion complexes of fullerenes with various macrocyclic hosts [21,22,23,24,25,26,27,28,29], the binding event is usually followed by UV-Vis and/or NMR spectroscopies, which normally require high concentrations of guests/hosts in solution for a reliable determination of the binding parameters. Fluorescence signaling of host–guest binding is much scarcer. Based on their previous work of fullerene encapsulation by calix[5]arenes [30], Fukazawa et al. developed the first C_60_/C_70_ luminescent sensor by appending a 2,2’-bipyridil-rhenium complex luminophore to a biscalix[5]arene unit [31]. Kawase and Oda et al. explored the use of carbon nanorings (cyclic [n]*p*-phenyleneethynylenes) for the complexation of C_60_/C_70_ [32]. Later, a series of poly(*p*-phenyleneethynylenes), differing in their molecular weight and side chain substituents, were used by Bucknall et al. to sense C_60_ [33]. Cyclotriveratrylene containing an appended array of carbazole units was studied by Shuang et al. for C_60_ detection [34]. Quenching of emission of a pillar[5]arene by C_60_, having attached sugar moieties at both rims, has also been investigated by Chen et al. [35]. More recently, Vigalok et al. conceived the usage of a 5,5′-bis-*p-tert*-butyl-calix[4]arene scaffold, fused at their phenolic *para* positions [36] and linked to an extended π-conjugated system (two phenyleneethynylene moieties), as a fluorescence-based sensor for C_70_ [37].

**Scheme 1:**
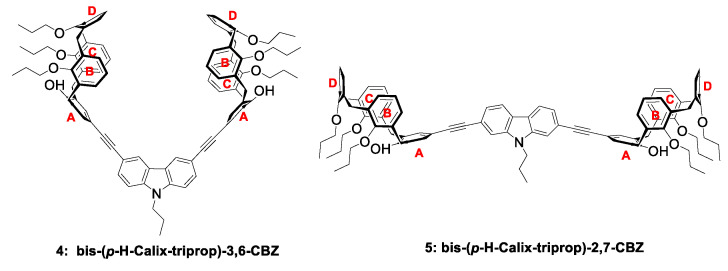
Chemical structures of bis-(*p-H*-Calix-triprop)-3,6-CBZ (**4**) and bis-(*p-H*-Calix-triprop)-2,7-CBZ (**5**).

Guided by supramolecular concepts—namely, the possible development of CH-π and π-π and dispersion interactions between calix[4]arene hosts and fullerenes C_60_ and C_70_, acting in combination with an ethynylene-carbazole luminescent signalling unit, we have designed new bis-calix[4]arene-carbazole conjugates (Scheme 1). Preliminary molecular mechanics force field (MMFF94) calculations have indicated that the diameter of the cavity formed by the two calixarene units and the carbazole moiety in the putative compound **4** would allow the formation of inclusion complexes with both fullerenes (C_60_ and C_70_) based on their sizes. On the contrary, specific interactions between **5** and those fullerenes would be unlikely to develop owing to geometric mismatch. Here, we describe the synthesis of novel fluorescent calixarenes **4** and **5** and complexation studies with fullerenes. Evidence for the formation of true inclusion complexes will be retrieved from NMR spectroscopy, in addition to steady-state and time-resolved fluorescence spectroscopies. The main conclusions will be assisted by computational methods using density functional theory (DFT) and semi-empirical quantum chemical calculations performed on the host–guest structures.

## 2. Results and Discussion

### 2.1. Synthesis and Structural Characterization

**Scheme 2:**
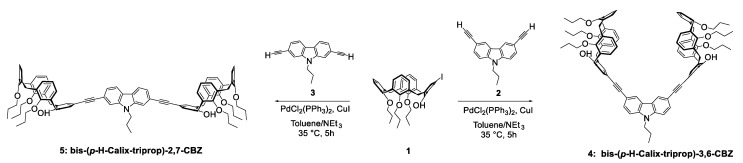
Cross-coupling of calix[4]arene-tripropyl-mono-iodo (**1**) and diethynyl-9-propyl-9*H*-carbazole derivatives (3,6-CBZ (**2**) and 2,7-CBZ (**3**)).

Fluorescent calix[4]arene-carbazole conjugates **4** and **5** were synthesized according to Scheme 2. calix[4]arene-tripropyl-mono-iodo derivative (**1**) was obtained by selective mono-iodination of the parent compound by adapting a reported procedure [38] (see Supplementary Materials (SM) for synthetic details and structural characterization). Pd-catalysed Sonogashira–Hagihara cross-coupling of **1** with 3,6-diethynyl-9-propyl-9*H*-carbazole (3,6-CBZ; **2**) [39] and 2,7-diethynyl-9-propyl-9*H*-carbazole (2,7-CBZ; **3**) [39], using PdCl_2_(PPh_3_)_2_ and CuI as a catalytic system in toluene under argon for 5 h at 35 °C, furnished the desired bis-calixarenes **4** and **5**, respectively. After flash chromatography, both compounds were isolated as yellow solids in low (7%; **5**) to moderate (37%; **4**) yields. It was found that, for a higher yield of the coupled product **4**, and cleaner reactions, the ethynyl-carbazole derivative **2** should be added very slowly (at least a 4 h dropwise addition) to **1**. In this way, the oxidative coupling of the ethynyl-carbazole units is minimized. Such procedure did not prove successful for **5**, yielding merely a 7% yield under these conditions. Looking for improved yields, microwave-assisted synthesis of **4** and **5** was tried. The reactions were carried at 35 °C for 1h, otherwise keeping the remaining experimental conditions. While, for **4,** a slight decrease in isolated product was observed (from 37% to 31%), a considerable improvement was accomplished for **5** (from 7% to 15%).

It is also worth mentioning that the success of the coupling reaction is strongly dependent on the aryl halide used. When a bromo derivative was used instead of the iodinated compound **1**, under a variety of experimental conditions-a large range of reaction temperatures and time, Pd(0) versus Pd(II) catalysts, and molar ratio of coupling partners-very complex reaction mixtures result and no useful yields of **4** or **5** could be retrieved.

Compounds **4** and **5** are freely soluble in CH_2_Cl_2_, CHCl_3_, THF, cyclohexane, and toluene.

The novel calix[4]arene-carbazole conjugates **4** and **5** were fully characterized through FTIR, ^1^H/^13^C NMR, and 2D NMR spectroscopies, and combi MALDI-ESI high-resolution mass spectrometry. All data are in excellent agreement with the proposed structures. Through extensive NMR analysis, using COSY, ^13^C DEPT 135, ^13^C-^1^H HSQC, ^13^C-^1^H HMBC, and NOESY techniques, a comprehensive H/C assignment has been made (cf. Experimental section and SM for details; numbering of H and aryl rings may be found in the insets of ^1^H NMR spectra).

A brief description of H assignments is highlighted below. The establishment of a cone conformation for the calixarene units was first undertaken, as they can adopt other plausible spatial arrangements through the flipping of aryl rings, in particular partial cone conformations. Compound **4** in CDCl_3_ displayed two sets of resonances for the bridged methylene groups in the calixarene skeleton: one at 3.22 (4H, d, *J* = 13.1 Hz) and 3.34 ppm (4H, d, *J* = 13.7 Hz) for equatorial protons and the other at 4.38 (4H, d, *J* = 13.9 Hz) and 4.42 ppm (4H, d, *J* = 13.2 Hz) for axial protons. The protons resonating at 3.22 ppm are coupled with those at 4.42 ppm, and these protons belong to the bridges linking ring A to rings B and C (cf. Scheme 1), while those at 3.34 and 4.38 ppm correspond to the other two bridges (ring D bridging with rings B and C). Such a disposition clearly indicates a cone conformation as the most stable arrangement of the calixarene units in **4**. For **5**, a similar attribution can be made, invoking the appearance of resonances at 3.23 and 3.34 ppm (d, 8H, *J* = 13.1 Hz and *J* = 13.8 Hz) and an unresolved multiplet between 4.34–4.49 ppm for equatorial and axial bridged methylene protons, respectively. In compound **4**, the multiplets at 6.32–6.45 (8H) and 6.46–6.56 ppm (4H) are assigned to protons on rings B and C, the triplet at 6.98 ppm (2H), and a doublet at 7.18 ppm (4H) to the resonances on ring D, and the multiplet at 7.35–7.46 ppm (4H) to those protons on ring A, which are overlapped with aryl protons (H_1_ and H_8_) of carbazole. Similar assignments for compound **5** may be found in the Experimental section. The complete set of NMR spectra of **4** and **5** are shown in Figures S1–S11 (SM).

The MALDI-ESI-HRMS spectra of compounds **4** and **5** are depicted in Figures S12 and S13.

### 2.2. Photophysical Properties

**Figure 1:**
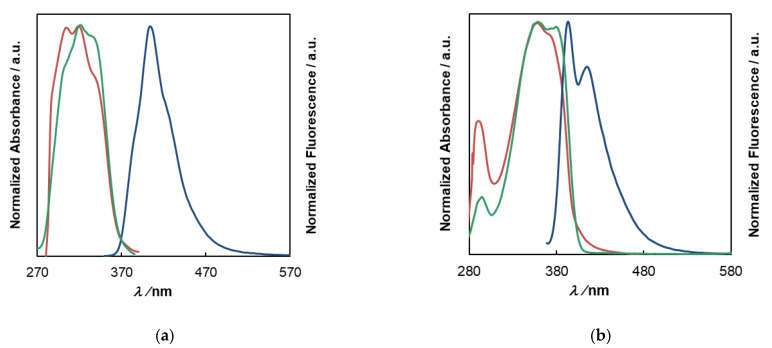
Absorption (red line), excitation (green line, monitored at emission maxima), and emission (blue line) spectra (*λ*_exc_ = 357 nm, toluene) of compounds **4** (**a**) and **5** (**b**).

The photophysical properties of bis-calixarenes **4** and **5** were studied by UV-Vis and steady-state and time-resolved fluorescence spectroscopies. Figure 1 depicts the ground-state absorption, excitation, and emission spectra of **4** and **5** in toluene solution, showing structured absorption and emission. UV-Vis (Figure S14) and steady-state emission (Figure S15) spectra of the compounds in various solvents (CH_2_Cl_2_, CHCl_3_, THF, cyclohexane, and toluene) are reported in SM. No significant solvatochromism was observed on any of these solvents either at ground- or excited-state, meaning that **4** and **5** are not very sensitive to solvent polarity.

The bis-calixarene-3,6-CBZ **4** exhibits several electronic transitions in the 270–380 nm region, being its absorption maximum around 319 nm, bearing a lower energy shoulder near 343 nm. The 2,7-CBZ isomer **5** displays the main absorption band peaking at 358 nm, accompanied by a shoulder at 377 nm. The extended π electron delocalization involving the bis-2,7-phenyleneethynylene-carbazole moiety in **5** is responsible for the observed red shifts (ca. 40 nm) of the main absorption band. The optical HOMO-LUMO energy gaps (*E*_g_), calculated from the low energy onset of the absorption bands of **4** and **5** (*E*_g(**4**)_ = 3.37 eV; *E*_g(**5**)_ = 3.08 eV) quantify the above observation (Table 1).

The emission spectra of both compounds showed different shapes, being both dominated by 0–0 transitions (*λ*_max_ at 404 and 394 nm for **4** and **5**, respectively). Vibronic progressions appear in both spectra at 424 and 414 nm for **4** and **5**, respectively, being highly noticeable for compound **5** (Figure 1b). Fluorescence quantum yields (*Φ*_F_) determined for compound **4** have shown to be strongly correlated with the solvent nature. Indeed, a deceptively low quantum yield (*Φ*_F_ = 0.028) was retrieved when CHCl_3_ was used as solvent; fortunately, the quantum yield raised significantly in other solvents (CH_2_Cl_2_, *Φ*_F_ = 0.13; THF, *Φ*_F_ = 0.18; cyclohexane, *Φ*_F_ = 0.20 and toluene, *Φ*_F_ = 0.17). No single correlation with the solvent polarity was found. As the selected solvent for inclusion studies with fullerenes was toluene, all the photophysical data involving **4** and **5** are presented in this solvent (Table 1), except where noted otherwise. It is of note that a much higher quantum yield was retrieved for **5** (*Φ*_F_ = 0.40).

**Figure 2:**
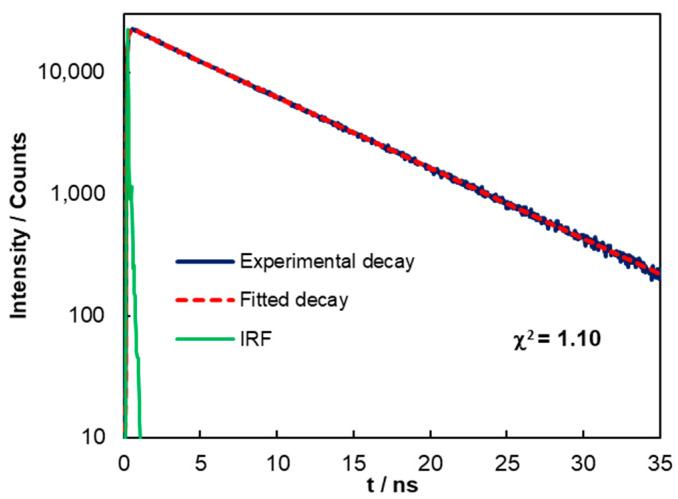
Time-resolved intensity decay of bis-calixarene-3,6-CBZ (**4**) in toluene obtained by the single-photon timing method under excitation at 340 nm.

The time-resolved fluorescence measurements were conducted by a single-photon timing method with laser excitation and microchannel plate detection under 340 nm excitation. The lifetime decay of **4** was best fitted by a sum of two exponentials (*τ*_1_ = 7.46 ns (99.1%) and *τ*_2_ = 1.63 ns (0.9%)), yielding an average lifetime (*τ*_ave_) of 7.40 ns in toluene solution (Figure 2).

A great stability toward photodegradation was observed for **4** and **5** (Figure S16a,b, respectively), under conditions of continuous irradiation (20 min) in toluene, ensuring a good starting point for fluorescence titration assays.

### 2.3. Complexation Studies with Fullerenes

**Figure 3:**
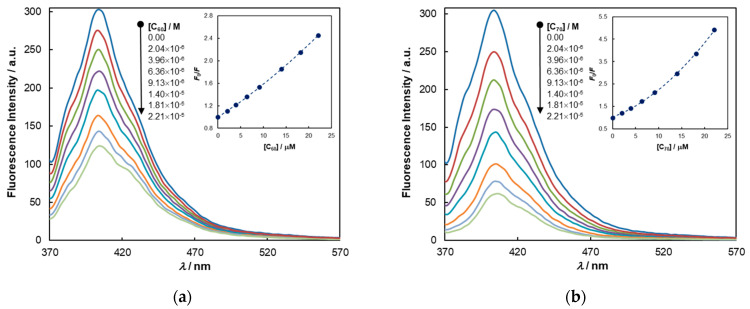
Emission spectra of bis-(*p*-H-calix-triprop)-3,6-CBZ (**4**) (6.0 × 10^−7^ M in toluene; *λ*_exc_ = 357 nm) after successive additions (2.04 × 10^−6^ – 2.21 × 10^−5^ M) of fullerene C_60_ (**a**) and C_70_ (**b**). Insets: plots of the changes in the ratio of fluorescence intensity of **4** vs. [C_60_] and [C_70_].

**Figure 4:**
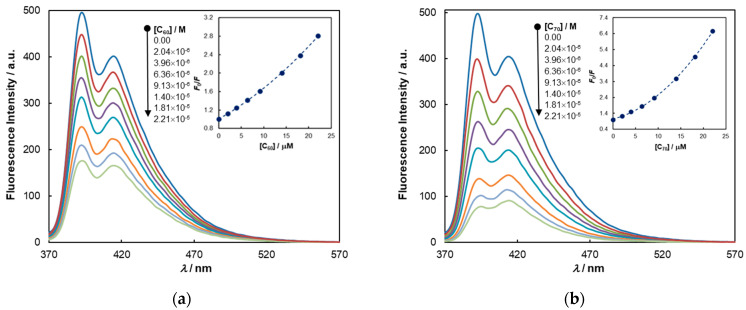
Emission spectra of bis-(*p*-H-calix-triprop)-2,7-CBZ (**5**) (6.0 × 10^−7^ M in toluene; *λ*_exc_ = 357 nm) after successive additions (2.04 × 10^−6^ – 2.21 × 10^−5^ M) of fullerene C_60_ (**a**) and C_70_ (**b**). Insets: plots of the changes in the ratio of fluorescence intensity of **5** vs. [C_60_] and [C_70_].

The design of the new bis-calixarene-carbazole conjugates **4** and **5** was directed toward the evaluation of their ability to form inclusion complexes with the fullerenes C_60_ and C_70_ in solution, in a sensitive and selective manner. The existence of any host–guest interactions was first assessed through fluorometric titrations in toluene. The experiments were performed with the guests in a concentration range of 2.04 × 10^−6^ – 2.21 × 10^−5^ M, while keeping that of the fluorophore constant (6.0 × 10^−7^ M) (Figure 3 and Figure 4). As is immediately perceived from the titration curves, a very strong quenching of the emission of the hosts **4** and **5** occur upon addition of C_60_ and C_70_. Plotting the ratio of the fluorescence intensities against the fullerene concentrations, curves showing an upward curvature are obtained. While this behavior can be associated with several quenching mechanisms - namely, a combined dynamic and static quenching mechanism and/or the existence of a sphere of effective quenching (i.e., a dynamic quenching within the sphere of action) [40] the high positive deviations from the linear Stern–Volmer (SV) equation are largely, in this case, simply due to hetero-inner-filter effects (h-IFEs).

Indeed, since both guests display, even at the low concentration range used, strong absorption of radiation at the excitation (357 nm) and emission wavelengths (404 nm for **4** and 394 nm for **5**) (see fullerenes absorption spectra in Figure S17), correction for h-IFEs is mandatory. Otherwise, huge errors may occur when calculating Stern–Volmer and/or association constants by fluorescence spectroscopy. Unfortunately, in many articles, including some related to the detection of fullerenes by fluorometric titration [41], no mention is given of this issue. The mathematical correction of fluorescence intensities was done according to ref. [42] (see Experimental section for details). The association constants (*K*_a_) of the supramolecular complexation (association or binding constants will be used interchangeably throughout the text) were calculated by a non-linear fitting approach (refer to Experimental section for calculation details) because no previous assumptions need to be made before applying it (for example, the existence, or not, of a non-fluorescent ground-state complex, or that the concentration of added guest is equal to the free guest), contrary to what occurs with the derived SV equation for static quenching or linearized forms of it (e.g., double reciprocal plots) [43,44]. The calculation of *K*_a_ by this methodology implies that dynamic quenching of the host fluorescence is absent or negligible. To verify this, time-resolved fluorescence assays were conducted with compound **4**. It is known that, in a static or in a sphere of effective quenching mechanism, the lifetimes of the fluorophores do not change upon increasing the concentration of the guests [40].

**Figure 5:**
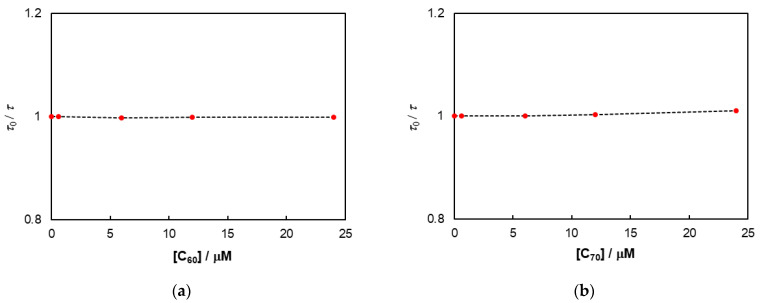
Lifetimes of **4** in toluene solution (6.0 × 10^−7^ M) upon addition (6.24 × 10^−7^ − 2.40 × 10^−5^ M) of fullerene C_60_ (**a**) and C_70_ (**b**) (*λ*_exc_ = 340 nm).

As may be clearly seen from Figure 5, a dynamic mechanism can be safely excluded in the quenching of **4′** fluorescence by fullerenes C_60_ and C_70_, since *τ*_0_/*τ* remains constant over the entire guest concentration range. A similar behavior was assumed to take place with **5**.

**Figure 6:**
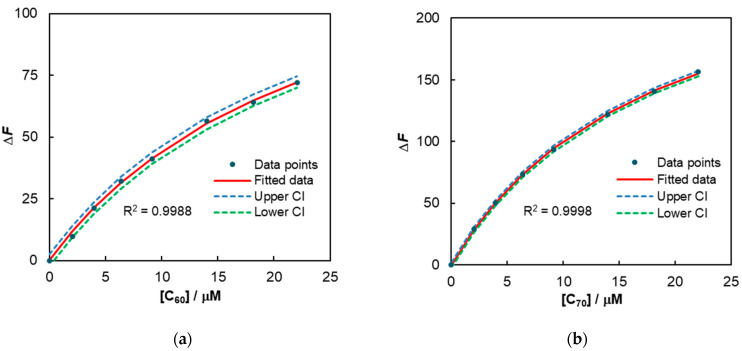

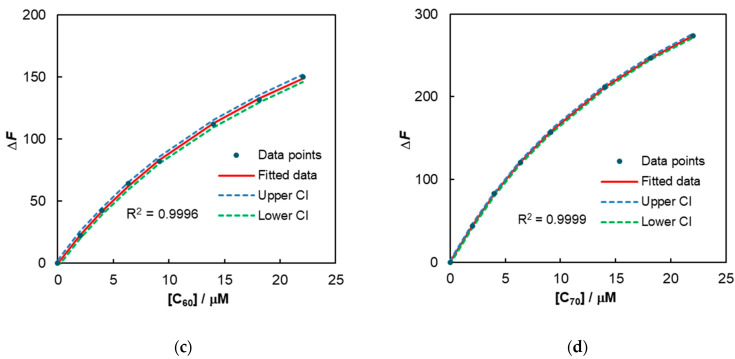
Binding isotherms for the fluorimetric titration of calixarene conjugates **4** (**a**,**b**) and **5** (**c**,**d**) with fullerene C_60_ and C_70_.

With this information at hand, the host–guest association constants were determined. The binding isotherms calculated for each host–guest pair using the non-linear regression are shown in Figure 6a–d together with the upper and lower confidence intervals (CI) for fitted data (cf. Experimental section).

Table 2 gathers the corresponding association constants and the estimated free energy of complexation (Δ*G*) associated with the binding event.

**Figure 7:**
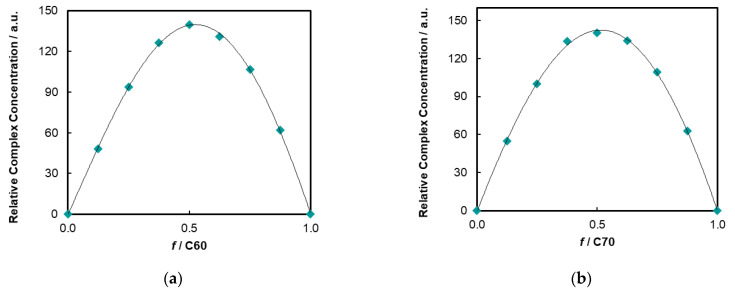
Job plot for complex formation between bis-calixarene-3,6-CBZ (**4**) and C_60_ (**a**) C_70_ (**b**) in toluene (at constant 6.0 × 10^−7^ M total concentration) as obtained from changes in fluorescence (*λ*_exc_ = 357 nm).

The excellent goodness of fit of the data by the non-linear approach assures that a 1:1 host–guest stoichiometry was attained in all cases. The continuous variation method (Job’s method) was additionally used to establish the stoichiometry of the complexes (see Experimental section for details). Figure 7 shows the Job plots of **4** with C_60_ and C_70_ (for **5**, see Figure S18). In either case, a maximum of the relative complex concentration is found at a mole fraction of 0.5 pointing to the formation of a 1:1 complex.

Comments on the above results are in order. First, all the binding constants are of significant magnitude (>3.7 × 10^4^ M^−1^), being the strongest binding observed between bis-calixarene-3,6-CBZ conjugate (**4**) and fullerene C_70_. This finding is in accordance with what was expected based on preliminary molecular mechanics force field (MMFF94) calculations [45] performed on the best supramolecular conformers of the host–guest pairs **4**: C_60_/C_70_, where a higher interaction energy was foreseen for C_70_ over C_60_ (ΔΔ*E* = −1.41 kcal/mol). These results were later fully corroborated by density functional theory (DFT) calculations (see below). Secondly, the considerable high association constants retrieved for compound **5** were somewhat surprising. The cavity of its structure, with a distance between the closest carbon atoms on the two D rings of 17.61 Å (comparing with 9.84 Å in **4**), seems to be too large to accommodate fullerene C_60_ (diameter of 7.05 Å) or C_70_ (7.94 Å across the larger axis of the ellipsoid) in a way that moderate to strong CH-π, π-π, and van der Walls interactions could be developed between the partners. One possibility to explain such results is to consider that unspecific π-π interactions between the electron rich phenyleneethynylene-derived carbazole unit and the electron-accepting fullerenes species, not related to the host–guest structural complementarity and, thus, not a true inclusion phenomenon, dominate the observed fluorescence quenching event. In other words, an apparent static quenching mechanism (sphere of effective quenching) may be in action. It is worth mentioning in this context that several PPEs devoid of molecular receptors in their structures were also able to sense C_60_ (*K*_SV_~1 × 10^4^ M^−1^), having the π-π interactions between the polymer backbone and C_60_ put forward to explain the results [33]. A 5,5′-bis-*p-tert*-butyl-calix[4]arene incorporating a phenyleneethynylene moiety, or its simple π-conjugated model, 4,4’-bis(phenylethynyl)-1,1’-biphenyl, showed even higher SV constants (*K*_SV_~2–3 × 10^4^ M^−1^) for C_70_ [37]; in this case, as the authors stated, no evidence was found for inclusion complexation.

From the above, we hypothesized that two types of quenching mechanisms may be involved on the fluorescence quenching of compound **4** with the fullerenes. One resulting from a sphere of effective quenching, resembling that observed with **5**, and the other a true static quenching mechanism stemming from the inclusion of C_60_/C_70_ in the host cavity. Heeger et al. have proposed a combination of static and sphere of action quenching mechanisms to explain the luminescence quenching of poly(phenylenevinylene) polyelectrolytes by methyl viologen [46].

Our attention was then turned to find experimental evidence for the existence of true inclusion complexes of **4** with C_60_/C_70_ and their possible spatial arrangements. For that, NMR experiments in toluene-d_8_ were undertaken. At room temperature (25 °C), the complexation-induced changes in the chemical shift (CIS) observed upon mixing equimolar amounts of **4** and C_60_ or C_70_ were very small. As the complex strength should increase at lower temperatures, variable-temperature (VT) NMR was performed for the host and for equimolar host–guest mixtures up to −80 °C (Figures S19–S21, supplementary materials), hoping that, in this way, enhanced CIS would result. The selected temperature for the observation of CIS was −10 °C since, at this temperature, a reasonable peak resolution is maintained. The CIS are, in general, of small magnitude (Table 3) but the reproducible downfield shifts observed for all the protons (calixarene and carbazole aryl protons) spatially closer to the fullerenes, which experience higher ring current effects, are consistent with the formation of inclusion complexes. Moreover, the AB sets of equatorial and axial protons at methylene bridges in **4** suffer a change in their shapes as a result of a conformational change upon complexation. Partial NMR spectra of representative regions are displayed in Figure S22.

**Figure 8:**
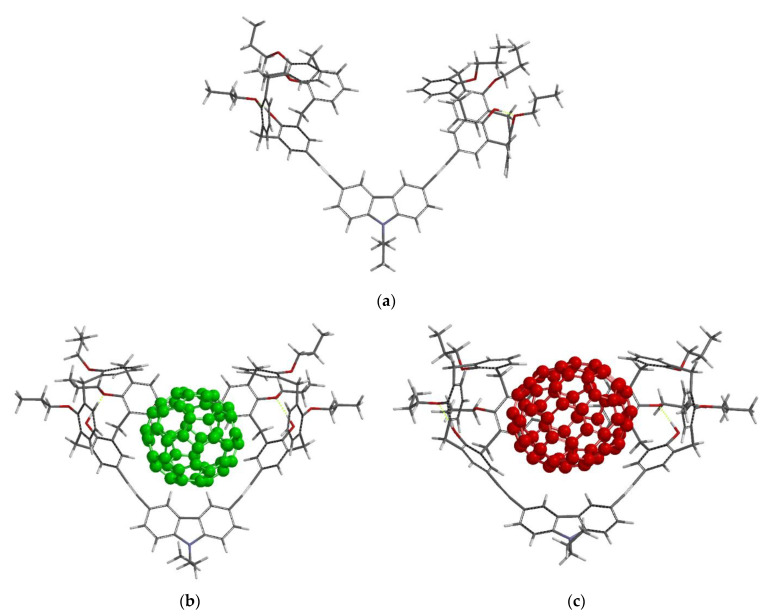
Bis-calixarene-3,6-CBZ (**4**) (**a**) and its supramolecular complexes with fullerenes C_60_ (**b**) and C_70_ (**c**). Geometry optimized structures were obtained by DFT calculations run at B3LYP/6-31G(d) level, and the energies calculated at D3(BJ)-B3LYP/6-311+G(2df,2p) level of theory in vacuum [45,47].

The foregoing experimental findings were very well corroborated by DFT calculations (for details see Experimental section) [45,47]. The two calixarene units in **4** adopt a pinched cone conformation, with the A and D rings almost parallel to each other and the rings B and C tilted outwards (Figure 8a); after inclusion of C_60_/C_70_ in the pocket of **4**, an almost symmetrical cone conformer results (Figure 8b,c). In the pinched cone, the O–O distance between distal rings A and D is 5.28 Å, and that of B and C rings is 4.21 Å (average distance in the two calixarene units); these turn, respectively, into 4.39 Å and 4.20 Å for C_60_@**4** and 4.43 Å and 4.29 Å for C_70_@**4**. One of the consequences would be the formation of stronger H-bonds as the average (two calixarene units) OH^…^OPr distance changes from 1.80 Å to 1.745 Å (both on C_60_ and C_70_); this probably reflects the significant downfield shifts observed for the phenolic protons in C_60_ (Δδ = 0.0099) and C_70_ (Δδ = 0.0178) assemblies, and the concomitant upfield shifts of *O*-methylenic protons on the propyl group of rings B, C, and D.

Analysis of the induced shifts reported in Table 3 clearly evidence that the protons of carbazole nucleus residing at positions 4,5 and 2,7 and those belonging to aryl calixarene units, particularly at the *meta* positions of bridged rings, suffer the highest deshielding effects by the presence of the fullerenes. This fact is more evident for C_70_@**4**, where the CIS are consistently higher than C_60_. Within the bis-calixarene-3,6-CBZ (**4**) pocket, closer contacts are expected between C_70_ and aryl protons, owing to its larger molecular size, as compared to C_60_, and the size of the (somewhat fixed) constructed cavity. This is supported by theoretical analysis (DFT) of host–guest distances. For C_70_, the average distance (in all cases, the two calixarene units are considered) of aryl protons (*meta* to CH_2_ bridges) on ring D to the closest carbon atom on the fullerene is 3.05 Å, where the same located proton on B/C rings is 3.28 Å; the average distance for the 4,5 protons on the carbazole is 3.165 Å. These values are comparable with 3.11 Å, 3.38 Å, and 3.105 Å reached by the same atoms on inclusion of C_60_. Fullerene-arene distances in various supramolecules are found in the range 3.0–3.5 Å [24].

The higher binding interactions of **4** with fullerene C_70_ observed in the NMR experiments are in qualitative agreement with the relative magnitudes of the association constants of **4** with C_60_/C_70_ obtained from fluorescence spectroscopy (Table 2).

The electronic binding energies of compound **4** and its inclusion complexes with C_60_ and C_70_ were calculated by high level DFT (see Experimental section for details of the methods) using recommended state-of-the-art procedures [48,49,50]. Dispersion-corrected functionals D3(BJ)-B3LYP and B97M-V were used in conjunction with an extended basis set (6-311+G(2df,2p)). The gas phase equilibrium binding energies (Δ*E*) were determined by the supramolecular approach using the equation: Δ*E* = *E*_complex_ − (*E*_host_ + *E*_guest_), where *E* corresponds to the total electronic energy of the species. The electronic binding energies of the complexes are presented in Table 4.

The results show that, in gas phase at 0 K, the supramolecule C70@**4** has a higher equilibrium electronic binding energy than C60@**4**, whatever the quantum mechanical model used in the calculations. These theoretical results (Δ*E*) are in line with those retrieved experimentally (Δ*G*; Table 2). For a direct comparison with the experimental results obtained in solution, the theoretical free energies of association (ΔG_th-sol_) in solution at, for example, 298 K, need to be calculated. The usual approach [48,49] is to consider it as a sum of three energy components: Δ*G*_th-sol_ = Δ*E* + Δ*G*_RRHO_ + ΔΔ*G*_solv_, where Δ*E* is the electronic binding energies in the gas phase, Δ*G*_RRHO_ is the gas phase free energy of the complex calculated in the rigid-rotor-harmonic oscillator approximation (sum of zero-point vibrational energy, temperature correction of enthalpy and entropy for each species in gas phase), and ΔΔ*G_solv_* is the change in the free energy of solvation upon complexation (ΔΔ*G*_solv_ = Δ*G*_solv-complex_ − (Δ*G*_solv-host_ + Δ*G*_solv-guest_)). Owing to the large size of our molecular species, Δ*G*_RRHO_ was calculated by the dispersion-corrected PM6-D3H4 semi-empirical method [51] as implemented in MOPAC 2016 [52] (see Experimental section for details). While the solvation free energy has been calculated for smaller systems using several advanced solvation models [53,54], not without criticism [29], huge computational resources and well-validated solvation models are needed for systems such as the ones treated here.

Nonetheless, an attempt was made to calculate the solvation contribution in our systems using the simpler Conductor-like Screening Model (COSMO) solvation model, also implemented in MOPAC (see Experimental section for details). The results are gathered in Table 5. There is a reasonable agreement between the experimentally found binding free energies for the fullerenes encapsulation by **4** and those obtained by the theoretical approaches. The theoretical results should, however, be taken with caution, since the methods used for the calculation of Δ*G*_RRHO_ and ΔΔ*G*_solv_, in particular the last contribution, contain errors. Further investigations are still needed to fully validate those results.

## 3. Materials and Methods

### 3.1. Instruments and Methods

FTIR were measured on a Bruker Vertex 70 spectrometer (Bruker Optik GmbH, Ettlingen, Germany) in transmission mode using KBr pellets. ^1^H NMR and ^13^C NMR spectra were collected on Bruker AVANCE II+ spectrometers (300.130 and 400.130 MHz) (Bruker BioSpin AG, Fällanden, Switzerland). The reported chemical shifts (δ/ppm) are internally referenced to CDCl_3_ (^1^H NMR, 7.26 ppm; ^13^C NMR, 77.16 ppm) and toluene (^1^H NMR, 2.09 ppm). The splitting parameters for ^1^H NMR are denoted as follows: s (singlet), d (doublet), t (triplet), dd (double doublet), sext (sextet), m (multiplet), and b (broad). ^13^C DEPT 135 (Distortionless Enhancement by Polarization Transfer), ^1^H-^1^H Correlation Spectroscopy (COSY), ^13^C-^1^H Heteronuclear Single Quantum Correlation (HSQC), Heteronuclear Multiple Bond Correlation (HMBC), and Nuclear Overhauser Effect Spectroscopy (NOESY) NMR techniques were used for spectral assignments [56]. All NMR spectra were recorded at room temperature (25 °C) except in the variable-temperature (VT) experiments (temperature range indicated in the stacked spectra).

High-resolution mass spectra were obtained at the Structural, Proteomic, and Xenomic Determination Services of Universidad de Vigo (C.A.C.T.I.) on a Solaris XR (FT-ICR) mass spectrometer (Bruker Daltonics, Billerica, MA, USA) equipped with a 7-Tesla actively shielded magnet. Ions were generated using a Combi MALDI-electrospray ionization (ESI) source. Ionization was achieved by electrospray, using a voltage of 4500 V applied to the needle, and a counter voltage of 300 V applied to the capillary. Samples were either prepared by adding a spray solution of 50:50 (*v/v*) Toluene/Acetone to a solution of the sample in toluene or a spray solution of 50:50 (*v/v*) CH_3_OH/Acetonitrile saturated with NaCl to a solution of the sample in CH_2_Cl_2_, at a *v/v* ratio of 1 to 5% to obtain the best signal-to-noise ratio. Data acquisition was performed using FtmsControl software version 2.2.0, and data processing was performed using DataAnalysis software, version 5.0, both from Bruker Daltonics.

UV-Vis spectra were recorded on a VWR UV 3100PC (VWR International bvba, Leuven, Belgium) or on a Jasco J-815 spectrophotometer (Jasco Inc., Tokyo, Japan) using 1 cm quartz cells at 25 °C.

Steady-state fluorescence spectra were collected on a PerkinElmer LS45 fluorimeter (PerkinElmer, Waltham, MA, USA) using a 1-cm quartz cuvette with right angle (RA) geometry at 25 °C in air-equilibrated conditions.

Time-resolved fluorescence intensity decay data was obtained by the single-photon timing method. The light source was a mode locked DPSS Nd:YVO4 green laser (Van-guard 2000-HM532, Spectra Physics) synchronously pumping a cavity dumped dye laser (701, Coherent, delivering frequency-doubled 3–4 ps pulses of about 40 nJ/pulse at 3.4 MHz) working with 4-(dicyanomethylene)-2-methyl-6-(4-dimethylaminostyryl)-4H-pyran (DCM). Emission light was detected by an Hamamatsu 2809U-01 microchannel plate photomultiplier. The excitation wavelength was 340 nm. Ensemble fluorescence decays data were analyzed with a sum of exponentials.

Fluorescence quantum yields were measured using 9,10-diphenylanthracene as fluorescence reference standard (*Φ* = 0.72, EtOH, air-equilibrated conditions, RA geometry) [57]. The quantum yields were determined by the slope method [58], keeping the optical densities of the sample and reference below 0.05 at the excitation wavelength to prevent inner filter effects.

Fluorimetric solution experiments were carried out by titration of diluted solutions (6 × 10^−7^ M) of fluorophores in toluene with known amounts of the analytes (fullerenes C_60_ and C_70_) using RA geometry. For each determination, at least three replicates were used. Correction of fluorescence intensities was done by the expression: *η* = Ax_0_Ay_0_(1 − 10^−Axi^)(1 −10^−Ay^_i_)/Ax_i_Ay_i_(1 − 10^−Ax^_0_)(1 − 10^−Ay^_0_), where Ax_0_ and Ay_0_ are the fluorophore absorbances and Ax_i_ = Ax_0_ + ΔAx_i_ and Ay_i_ = Ay_0_ + ΔAy_i_ are the total absorbances of the fluorophore and the quencher (ΔAx_i_ and ΔAy_i_) at the excitation and emission wavelengths, respectively [42]. On applying this correction, it is assumed that the fluorescence intensity comes exactly from the center of a centrally illuminated cuvette under RA observation.

The association constant (*K*_a_) for the formation of a supramolecular complex (HG) between the host (H) and the guest (G), from the 1:1 equilibrium H + G ⇄ HG, can be described by *K*_a_ = [HG]/[H][G]. It can be shown [59] that the association constant may be calculated by solving the following equation:

Δ*F* = ½{Δ*ε*_F_([H]_0_ + [F]_0_ + 1/*K*_a_) − [Δ*ε*_F_^2^([H]_0_ + [F]_0_ + 1/*K*_a_)^2^ − 4Δ*ε*_F_^2^[H]_0_[F]_0_]^1/2^}, where Δ*F* and Δ*ε*_F_ are the changes in fluorescence intensity and molar fluorescence intensity of the hosts upon complexation with fullerenes, *K*_a_ is the association constant, and [H]_0_ and [F]_0_ denote the initial concentrations of the host and the fullerene. Calculations were performed by a non-linear regression analysis using the Solver function in Microsoft Excel [60], with a non-linear generalized reduced gradient (GRG) algorithm. The upper and lower confidence intervals (CI) for fitted data were calculated from the standard error of residuals and the critical *t*-value [60].

The Job plots were obtained by plotting the concentration of the host–guest complexes ([HG]) against the mole fraction (*f*) of the guest (fullerene); the maximum of [HG] corresponds to mole fraction of the guest in the complex. The relative concentration of the complex was calculated from [HG] = (*F*_0_ − *F*)/*F*_0_ × [H], where (*F*_0_ − *F*)/*F*_0_ is the relative fluorescence intensities and [H] is the concentration of the host (**4** or **5**).

Reactions under microwave irradiation were performed by using a mono-mode microwave reactor (CEM, Discover), and pressure-rated reaction vials with poly(tetrafluoroethylene)-silicon caps.

### 3.2. Materials

calix[4]arene-tripropyl-mono-iodo derivative **1** (Scheme S1) was obtained by selective mono-iodination from 25-hydroxy-26,27,28-tripropoxycalix[4]arene [61] by an adapted synthetic procedure [38]; details of its synthesis and structural characterization can be found in SM [62,63,64]. Additionally, 3,6-diethynyl-9-propyl-9*H*-carbazole (**2**) and 2,7-diethynyl-9-propyl-9*H*-carbazole (**3**) [39] were synthesized according to our reported method. Both compounds were fully characterized by FTIR and NMR spectroscopies.

Dichlorobis(triphenylphosphine)palladium (II) (98%, Aldrich, Sigma-Aldrich Corp.,St. Louis, MO, USA), copper(I) iodide (98%, Aldrich, Sigma-Aldrich Corp.,St. Louis, MO, USA), iodobenzene (98%, Acros, NJ, USA), Fullerene-C_60_ (99.5%, Aldrich, Sigma-Aldrich Corp., St. Louis, MO, USA), Fullerene-C_70_ (98%, Aldrich, Sigma-Aldrich Corp.,St. Louis, MO, USA), and 9,10-diphenylanthracene (scintillation grade, Nuclear Enterprises Ltd., Edinburgh, UK) were used as received. Triethylamine (99%, Riedel-de-Haën, Honeywell, Seelze, Germany) was previously dried from CaH_2_ and distilled under N_2_ prior to use. *p*-Toluenesulfonic acid (98.5%, Sigma-Aldrich, Sigma-Aldrich Corp.,St. Louis, MO, USA) was recrystallized from chloroform and *N*-iodosuccinimide (NIS; 97%, Alfa Aesar, Massachusetts, USA) was recrystallized from dioxane/carbon tetrachloride. Toluene was previous dried from Na, distilled under N_2_, and stored over Na. All other reagents and solvents were reagent grade and were purified and dried by standard methods. Organic extracts were dried over anhydrous magnesium sulphate. Analytical thin-layer chromatography (TLC) was carried out on E. Merck Kieselgel 60 (Merck, Darmstadt, Germany), F-254 silica-gel 0.2 mm thick plates. Flash column chromatography was performed on E. Merck Kieselgel 60 (230–400 mm) silica gel (Merck, Darmstadt, Germany).

### 3.3. Synthesis

Compound **4**: To an argon degassed solution containing 120 mg (177.0 µmol) of **1** [38] in freshly distilled NEt_3_ (7.1 mL) were added PdCl_2_(PPh_3_)_2_ (8.9 mg, 12.4 µmol) and CuI (2.41 mg, 12.4 µmol). The yellow suspension was degassed, the flask sealed, and the contents stirred in a pre-heated bath at 35 °C. A second solution of 3,6-carbazole monomer **2** (21.7 mg, 85.0 µmol) [39] in dry and degassed toluene (7.1 mL) was added dropwise to the calixarene solution for 4 h and allowed to react for an additional hour. The mixture acquired a yellow color that gradually turned red-orangish with turbidity. The reaction was followed by TLC [AcOEt:Hexane (1:8)], which, after 5 h, revealed the consumption of **1**. Solvents were removed by evaporation and the residue taken in CH_2_Cl_2_ and washed successively with aqueous solutions of 2% HCl, 0.1M NaHSO_3_, 10% NH_4_SCN, and water. The organic extract was dried and evaporated to dryness. After flash chromatograph [AcOEt:Hexane (1:8)], compound **4** was isolated as a light-yellow solid in 37% (42.6 mg). The procedure using microwave-assisted synthesis was carried out at 35 °C during 1h under stirring, yielding 31% of **4**. *m.p.*: 186–188 °C. *v*_max_/cm^−1^ (film) 3533, 3059, 3032, 3018, 2963, 2920, 2875, 2203, 1626, 1590, 1568, 1488, 1458, 1434, 1384, 1350, 1285, 1248, 1223, 1204, 1152, 1129, 1108, 1086, 1068, 1041, 1004, 964, 881, 843, 806, 762, 738, 630; *λ*_max_/nm (*ε*_max_ × 10^−4^ M^−1^ cm^−1^) 304 (4.70), 319 (4.73), 343 (sh, 3.42), cutoff at 420 nm. *δ*_H_ (CDCl_3_, 400.130 MHz) 0.94 (t, 6H, -*O*-CH_2_-CH_2_-CH_3_, *J* = 7.5 Hz, D rings), 1.01 (t, 3H, -*N*-CH_2_-CH_2_-CH_3_, *J* = 7.4 Hz), 1.13 (t, 12H, -*O*-CH_2_-CH_2_-CH_3_, *J* = 7.4 Hz, B and C rings), 1.82-2.03 (m, 10H, -*O*-CH_2_-CH_2_-CH_3_ (8H), B and C rings, and -*N*-CH_2_-CH_2_-CH_3_ (2H)), 2.21–2.37 (m, 4H, -*O*-CH_2_-CH_2_-CH_3_, D rings), 3.22 (d, 4H, ArCH_2eq_Ar, *J* = 13.1 Hz), 3.34 (d, 4H, ArCH_2eq_Ar, *J* = 13.7 Hz), 3.67–3.82 (m, 8H, -*O*-CH_2_-CH_2_-CH_3_, B and C rings), 3.82–3.92 (m, 4H, -*O*-CH_2_-CH_2_-CH_3_, D rings), 4.29 (t, 2H, -*N*-CH_2_-CH_2_-CH_3_, *J* = 7.3 Hz), 4.38 (d, 4H, ArCH_2ax_Ar, *J* = 13.9 Hz), 4.42 (d, 4H, ArCH_2ax_Ar, *J* = 13.2 Hz), 5.08 (s, 2H, ArOH), 6.32–6.45 and 6.46–6.56 (m, 12H, Ar_Calix_H, B and C rings), 6.98 (t, 2H, Ar_Calix_H, *J* = 7.4 Hz, D rings), 7.18 (d, 4H, Ar_Calix_H, *J* = 7.5 Hz, D rings), 7.35–7.46 (m, 6H, Ar_Calix_H (4H), A rings and Ar_CBZ_H_(1,8)_ (2H)), 7.67 (dd, 2H, Ar_CBZ_H_(2,7)_, *J_o_* = 8.5 Hz, *J_m_* = 1.7 Hz), 8.30 (bs, 2H, Ar_CBZ_H_(4,5)_); *δ*_C_ (CDCl_3_, 75.468 MHz) 9.72 (-*O*-CH_2_-CH_2_-CH_3_, D rings), 10.97 (-*O*-CH_2_-CH_2_-CH_3_, B and C rings), 11.96 (-*N*-CH_2_-CH_2_-CH_3_), 22.52 (-*O*-CH_2_-CH_2_-CH_3_, D rings), 23.58 (-*O*-CH_2_-CH_2_-CH_3_, B and C rings and -*N*-CH_2_-CH_2_-CH_3_), 30.71, 30.87 (ArCH_2_Ar), 44.87 (-*N*-CH_2_-CH_2_-CH_3_), 76.47, 77.55 (-*O*-CH_2_-CH_2_-CH_3_), 88.60, 88.70 (-C≡C-), 109.09, 109.11 (Ar_CBZ_C_(1,8)_ and Ar_Calix_C), 114.12 (Ar_CBZ_C_(3,6)_), 114.54 (Ar_CBZ_C_(4a,4b)_), 122.68, 123.16 (Ar_Calix_C), 123.26, 124.00 (Ar_CBZ_C_(4,5)_), 128.08, 128.22, 129.31 (Ar_Calix_C), 129.69 (Ar_CBZ_C_(2,7)_), 131.10 (Ar_Calix_C(OH)), 131.94 (Ar_Calix_C), 132.19, 133.57, 137.26 (Ar_Calix_C-CH_2_-), 140.40 (Ar_CBZ_C_(8a,9a)_), 153.89, 154.41, 156.98 (Ar_Calix_C-O-CH_2_-CH_2-_CH_3_). ^13^C DEPT 135, COSY, ^13^C-^1^H HSQC, ^13^C-^1^H HMBC, and NOESY NMR experiments (CDCl_3_) were used for the spectral assignments (Figures S1–S7). ESI-HRMS: *m/z* calcd for C_93_H_96_NO_8_^+^: 1354.7136 [M + H]^+^; found: 1354.7127 (Figure S12).

Compound **5**: The same procedure, using calixarene **1** (166 mg, 245 μmol) [38], freshly distilled NEt_3_ (9.8 mL), PdCl_2_(PPh_3_)_2_ (12.3 mg, 17.1 μmol), CuI (3.33 mg, 17.1 μmol), and 2,7-carbazole monomer **3** (60.0 mg, 233 µmol) [39] in dry toluene (9.8 mL) afforded 10.7 mg (7%) of calixarene **5** as a bright yellow solid. Using microwave irradiation during 1 h at 35 °C, **5** was isolated in 15%. *m.p.*: 223–226 °C (dec.). *v*_max_/cm^−1^ (film) 3534, 3060, 3031, 3018, 2960, 2924, 2874, 2854, 2204, 2083, 1651, 1624, 1598, 1558, 1481, 1458, 1438, 1386, 1328, 1295, 1249, 1229, 1208, 1200, 1161, 1106, 1086, 1073, 1065, 1042, 1004, 965, 907, 881, 847, 807, 763, 739, 633; *λ*_max_/nm (*ε*_max_ × 10^−4^ M^−1^ cm^−1^) 294 (2.33), 358 (4.18), 377 (3.81) cutoff at 460 nm. *δ*_H_ (CDCl_3_, 300.130 MHz) 0.95 (t, 6H, -*O*-CH_2_-CH_2_-CH_3_, *J* = 7.4 Hz, D rings), 1.04 (t, 3H, -*N*-CH_2_-CH_2_-CH_3_, *J* = 7.4 Hz), 1.13 (t, 12H, -*O*-CH_2_-CH_2_-CH_3_, *J* = 7.4 Hz, B and C rings), 1.82–2.04 (m, 10H, -*O*-CH_2_-CH_2_-CH_3_ (8H), B and C rings and -*N*-CH_2_-CH_2_-CH_3_ (2H)), 2.23–2.36 (m, 4H, -*O*-CH_2_-CH_2_-CH_3_, D rings), 3.23 (d, 4H, ArCH_2eq_Ar, *J* = 13.1 Hz), 3.34 (d, 4H, ArCH_2eq_Ar, *J* = 13.8 Hz), 3.76 (t, 8H, -*O*-CH_2_-CH_2_-CH_3_, *J* = 6.5 Hz B and C rings), 3.85 (t, 4H, -*O*-CH_2_-CH_2_-CH_3_, *J* = 8.4 Hz D rings), 4.29 (t, 2H, -*N*-CH_2_-CH_2_-CH_3_, *J* = 7.1 Hz), 4.33–4.49 (m, 8H, ArCH_2ax_Ar), 5.20 (s, 2H, ArOH), 6.33–6.53 (m, 12H, Ar_Calix_H, B and C rings), 6.99 (t, 2H, Ar_Calix_H, *J* = 7.4 Hz, D rings), 7.19 (d, 4H, Ar_Calix_H, *J* = 7.4 Hz, D rings), 7.40 (s, 4H, Ar_Calix_H, A rings), 7.43 (d, 2H, *J* = 8.3 Hz, Ar_CBZ_H_(3,6)_), 7.61 (s, 2H, Ar_CBZ_H_(1,8)_), 8.04 (d, 2H, Ar_CBZ_H_(4,5)_, *J* = 8.1 Hz); *δ*_C_ (CDCl_3_, 75.468 MHz) 9.72 (-*O*-CH_2_-CH_2_-CH_3_, D rings), 10.97 (-*O*-CH_2_-CH_2_-CH_3_, B and C rings), 11.95 (-*N*-CH_2_-CH_2_-CH_3_), 22.44, 22.53 (-*O*-CH_2_-CH_2_-CH_3_, D rings), 23.58, 29.85 (-*O*-CH_2_-CH_2_-CH_3_, B and C rings and -*N*-CH_2_-CH_2_-CH_3_), 30.70, 30.86 (ArCH_2_Ar), 44.94 (-*N*-CH_2_-CH_2_-CH_3_), 77.78 (-*O*-CH_2_-CH_2_-CH_3_), 89.03, 90.25 (-C≡C-), 111.94 (Ar_CBZ_C_(1,8)_), 113.71 (Ar_CBZ_C_(4a,4b)_), 120.47, 121.10 (Ar_CBZ_C_(4,5)_), 122.40 (Ar_CBZ_C_(3,6)_), 122.89, 123.18, 123.27, 128.02, 128.03, 128.07, 129.32, 131.10, 132.10, 133.61, 137.23 (Ar_Calix_C), 140.96 (Ar_CBZ_C_(8a,9a)_), 154.17, 154.40, 156.97 (Ar_Calix_C-O-CH_2_-CH_2-_CH_3_). ^13^C DEPT 135, COSY, and ^13^C-^1^H HSQC NMR experiments (CDCl_3_) were used for the spectral assignments (Figures S8–S11). ESI-HRMS: *m*/*z* calcd for C_93_H_95_NNaO_8_^+^: 1376.6955 [M + Na]^+^; found: 1376.6943 (Figure S13).

### 3.4. Computational Methods

The best (lowest energy) conformers of bis-calixarene-3,6-CBZ (**4**) and its complexes with C_60_ and C_70_ were searched by the Monte Carlo method, and their geometries optimized using a molecular mechanics force field (MMFF94). Density functional theory (DFT) calculations running on a hybrid model (B3LYP) with a small 6-31G(d) basis set were then performed for geometry optimization. Dispersion-corrected functionals were further used for single-point energy calculations. A global hybrid generalized gradient approximation (GGA) functional (D3(BJ)-B3LYP), with two-body and three-body dispersion energy terms and Becke-Johnson damping, and a local meta GGA (B97M-V) functional were used for this purpose, in combination with an extended basis set (6-311+G(2df,2p)).

All quantum mechanics calculations were performed by Q-Chem 5.1 [47] running on Spartan’18 molecular modelling software [45] using default integration grids (SG0/SG-1 for B3LYP and (75, 302) for B97M-V) and convergence criteria, in vacuum. The selection of the functionals for the study was based on the well-documented behavior of their performance in systems associated with non-covalent interactions [50].

The dispersion-corrected PM6-D3H4 semi-empirical method [51], as implemented in MOPAC 2016 version: 21.002W [52], was used for the calculation of thermodynamic parameters used in Δ*G*_RRHO_ calculations, and solvation energies. Input geometries from B3LYP/6-31G(d) were fully optimized using the L-BFGS geometry optimizer and PRECISE keyword (see MOPAC manual for details at http://openmopac.net/manual/index.html). The thermodynamic quantities were obtained with the THERMO keyword at default temperature (298 K). No corrections were applied to the anharmonicities associated with the thermal contributions from the low-frequency vibrational modes (< 100 cm^−1^) during the Δ*G*_RRHO_ calculations. The COSMO solvation energies were calculated by the MOPAC formalism, using a dielectric constant of 2.38 correspondent to toluene.

## 4. Conclusions

Based on simple principles of host–guest chemistry, two new fluorescent bis-calix[4]arene-carbazole conjugates **4** and **5** were designed to function as hosts for fullerenes C_60_ and C_70_. The syntheses of **4** and **5** were accomplished in a one-pot double cross-coupling Sonogashira–Hagihara reaction, with **4** having been isolated in synthetic useful yields. The oxidative coupling of 3,6-diethynyl-9-propyl-9*H*-carbazole, or its 2,7-diethynyl isomer, leading to oligomeric/polymeric structures, is the major competitive reaction. Full structural characterization of **4** and **5** was undertaken (^1^H/^13^C NMR, 2D NMR, and ESI-HRMS), and their photophysical properties thoroughly evaluated (UV-Vis, and steady-state/time-resolved fluorescence spectroscopies).

The complexation of C_60_ and C_70_ by **4** and **5** was followed by fluorometric titrations in toluene solution. A great level of fluorescence quenching was observed for both hosts. Time-resolved fluorescence titrations with compound **4** and both fullerenes have indicated that dynamic quenching (occurring during the lifetime of excited host) was absent. This allowed the calculation of the association constants of the binding events by a non-linear fitting approach. It was found that, in toluene, **4** binds preferentially with C_70_ over C_60_ in a 1:1 stoichiometry, yielding, in either case, stable complexes (Δ*G* = −6.48 and −6.31 kcal/mol, respectively). Surprisingly, high association constants were also obtained for **5** on interaction with C_60_/C_70_. The observed quenching was interpreted, in this case, as being largely a result of a sphere of effective quenching mechanism, since structural complementarity between **5** and the fullerenes barely exist, considering the geometrical mismatch between the dimension of the nanocavity of **5** and the size of the guests. In the case of host **4**, evidence for the formation of true inclusion complexes (C_60_@**4** and C_70_@**4**) came from low-temperature NMR assays. Theoretical calculations performed on the complexes at DFT level are in excellent agreement with the experimental findings.

The bis-calix[4]arene-carbazole conjugates **4** and **5** here disclosed are now being investigated as hosts for the construction of other supramolecular systems.

## Figures and Tables

**Table 1:** Photophysical properties ^1^ of bis-calixarene-carbazole conjugates.

Compound	*λ*_abs max_/nm (log*ε*_max_)	*E*_g_^2^/eV	*λ*_em max_/nm	Stokes Shift ^3^/nm	*Φ*_F_^4^
4	319 (4.675)	3.37	404	85	0.17
5	358 (4.621)	3.08	394	36	0.40

^1^ All data was collected using toluene as solvent; ^2^ optical energy gaps (*E*_g_) were calculated from the low energy onset of the absorption bands; ^3^ Stokes shifts were calculated from *λ*_F,max_ (0–0) − *λ*_A,max_; ^4^ fluorescence quantum yields were determined using 9,10-diphenylanthracene as reference in air-equilibrated conditions at *λ*_exc_ = 340 nm.

**Table 2:** Association constants (*K*_a_) and free energy changes (Δ*G*) for complex formation with fullerenes.

	C_60_		C_70_	
Host	*K*_a_/M^−1^	Δ*G* ^1^/kcal/mol	*K*_a_/M^−1^	Δ*G* ^1^/kcal/mol
**4**	42,400 ± 3500 (R^2^ = 0.9988)	−6.31	55,800 ± 1800 (R^2^ = 0.9998)	−6.48
**5**	36,800 ± 1900 (R^2^ = 0.9996)	−6.23	44,000 ± 900 (R^2^ = 0.9999)	−6.33

^1^ Determined at 298.15 K.

**Table 3:** Complexation-induced changes in the chemical shift (CIS) ^1,2^ of selected protons on **4** in toluene-d_8_ solution.

	C_60_@4	C_70_ @4
H Location	Δδ/ppm	Δδ/ppm
CBZ_4,5_	0.0020	0.0043
CBZ_2,7_	0.0022	0.0066
Calix_ring A_	0.0017	0.0036
CBZ_1,8_	^3^	−0.0056
Calix_rings B, C_^4^	0.0071	0.0091
Calix_ring A-phenolic_	0.0099	0.0178
Calix_ring D-OCH2CH2CH3_	−0.0040	−0.0040
CBZ_-OCH2CH2CH3_	−0.0030	−0.0040
Calix_ring B, C-OCH2CH2CH3_	−0.0027	−0.0034

^1^ Determined at −10 °C; ^2^ CIS were calculated by Δδ = δcomplex − δfree host; ^3^ signals overlapped with solvent; ^4^ complex pattern; all the protons in the range 6.42–6.60 ppm move downfield; the largest Δδ observed, and indicated in the table, corresponds to the signal appearing at 6.47 ppm in **4**.

**Table 4:** Electronic binding energies ^1^ for complexation of **4** with fullerenes.

	D3(BJ)-B3LYP Functional	B97M-V Functional
Complex	Δ*E*/kcal/mol	Δ*E*/kcal/mol
C60@**4**	−36.92	−39.31
C70@**4**	−39.28	−42.92
ΔΔ*E*	−2.36	−3.60

^1^ Determined by DFT calculations using the above functionals with 6-311+G(2df,2p) basis set in gas phase at 0 K [45,47].

**Table 5:** Theoretical binding free energies (Δ*G*_th-sol_) for complexation of **4** with fullerenes in toluene at 298 K.

Complex	Δ*E* ^1^/ kcal/mol	Δ*G*_RRHO_^2^/ kcal/mol	Δ*G*_1M_^3^/ kcal/mol	ΔΔ*G*_solv_^4^/ kcal/mol	Δ*G*_th-sol_/ kcal/mol
C_60_@**4**	−39.31	+29.57	−11.64	+5.74	−5.9
C_70_@**4**	−42.92	+32.09	−12.73	+6.00	−6.7

^1^ From DFT calculations at B97M-V/6-311+G(2df,2p) level [45,47]; ^2^ calculated with PM6-D3H4 semi-empirical method at 298 K [47]; ^3^ the free energy Δ*G*_1M_ for the standard state in solution (1M) is obtained from free energy Δ*G*_g_ for the standard state in gas phase (1 atm) (Δ*G*_g_ = Δ*E* + Δ*G*_RRHO_) by subtracting the amount RTln(RT/P) [55], which equals + 1.902 kcal/mol at 298 K; ^4^ calculated by COSMO solvation model using toluene as solvent [52].

## Data Availability

The spectral data presented are available on request from the corresponding author.

## Supplementary Materials

The following are available online: Experimental details of the synthesis and characterization of compound **1** (Scheme S1); ^1^H/^13^C NMR and 2D NMR spectra of compound **4** (Figures S1–S7); ^1^H/^13^C NMR and 2D NMR spectra of compound **5** (Figures S8-S11); ESI-HRMS spectra of compound **4** (Figure S12) and compound **5** (Figure S13); UV-Vis spectra of compounds **4** and **5** in various solvents (Figure S14); Emission spectra of compounds **4** and **5** in various solvents (Figure S15); Emission spectra of compounds **4** and **5** upon continuous irradiation (Figure S16); UV-Vis spectra of fullerenes C_60_ and C_70_ (Figure S17); Job plots of complex formation between compound **5** and C_60_/C_70_; VT-^1^H NMR spectra of compound **4** (Figure S19); VT-^1^H NMR spectra of C_60_@**4** (Figure S20); VT-^1^H NMR spectra of C_70_@**4** (Figure S21); Stacked partial ^1^H NMR spectra of compound **4**, C_60_@**4**, and C_70_@**4** at −10 °C (Figure S22).

## Sample Availability

Samples of the compounds are not available from the authors.
