# Supplementary material for: Fluorescent Bis-Calix[4]arene-Carbazole Conjugates: Synthesis and Inclusion Complexation Studies with Fullerenes C60 and C70
Source: Molecules. 2021 Aug 18;26(16):5000. doi: 10.3390/molecules26165000 (PMC8399125; doi:10.3390/molecules26165000)
Supplement: Supplementary file 1 [file molecules-26-05000-s001.zip › Molecules-1337182-SM.pdf]

molecules

MDPI

Article

Fluorescent Bis-Calix[4]arene-Carbazole Conjugates: Synthesis and Inclusion Complexation Studies with Fullerenes C_{60} and C_{70}

Patrícia D. Barata [1,2], Alexandra I. Costa [1,2], Sérgio Costa [1], José V. Prata [1,2,*]

[1] Departamento de Engenharia Química, Instituto Superior de Engenharia de Lisboa, Instituto Politécnico de Lisboa, R. Conselheiro Emídio Navarro, 1, 1959-007 Lisboa, Portugal. acosta@deq.isel.ipl.pt (A.I.C.); pbarata@deq.isel.ipl.pt (P.D.B.); sethcosta@hotmail.com (S.C.)

[2] Centro de Química-Vila Real, Universidade de Trás-os-Montes e Alto Douro, 5001-801 Vila Real, Portugal

* Correspondence: jvprata@deq.isel.ipl.pt (J.V.P.); Tel.: +351-218317172

Supplementary Materials

Table of Contents

Scheme S1. Synthesis of calix[4]arene-tripropyl-mono-iodo derivative **1**.

Figure S1. ^{1}H NMR spectrum of bis-(*p*-H-calix-triprop)-3,6-CBZ (**4**).

Figure S2. ^{13}C NMR spectrum of bis-(*p*-H-calix-triprop)-3,6-CBZ (**4**).

Figure S3. ^{13}C DEPT 135 NMR spectrum of bis-(*p*-H-calix-triprop)-3,6-CBZ (**4**).

Figure S4. ^{1}H-^{1}H COSY NMR spectrum of bis-(*p*-H-calix-triprop)-3,6-CBZ (**4**).

Figure S5. ^{1}H-^{13}C HSQC NMR spectrum of bis-(*p*-H-calix-triprop)-3,6-CBZ (**4**).

Figure S6. ^{1}H-^{13}C HMBC NMR spectrum of bis-(*p*-H-calix-triprop)-3,6-CBZ (**4**).

Figure S7. NOESY NMR spectrum of bis-(*p*-H-calix-triprop)-3,6-CBZ (**4**).

Figure S8. ^{1}H NMR spectrum of bis-(*p*-H-calix-triprop)-2,7-CBZ (**5**).

Figure S9. ^{13}C NMR (**a**) and ^{13}C DEPT 135 NMR (**b**) spectra of bis-(*p*-H-calix-triprop)-2,7-CBZ (**5**).

Figure S10. ^{1}H-^{1}H COSY NMR spectrum of bis-(*p*-H-calix-triprop)-2,7-CBZ (**5**).

Figure S11. ^{1}H-^{13}C HSQC NMR spectrum of bis-(*p*-H-calix-triprop)-2,7-CBZ (**5**).

Figure S12. Full ESI-HRMS spectrum of bis-(*p*-H-calix-triprop)-3,6-CBZ (**4**) (**a**); ESI-HRMS spectrum of the molecular ion region (**b**).

Figure S13. Full ESI-HRMS spectrum of bis-(*p*-H-calix-triprop)-2,7-CBZ (**5**) (**a**); ESI-HRMS spectrum of the molecular ion region (**b**).

Figure S14. UV-Vis spectra of bis-(*p*-H-calix-triprop)-3,6-CBZ (**4**) and bis-(*p*-H-calix-triprop)-2,7-CBZ (**5**) in various solvents.

Figure S15. Steady-state emission spectra of bis-(*p*-H-calix-triprop)-3,6-CBZ (**4**) and bis-(*p*-H-calix-triprop)-2,7-CBZ (**5**) in various solvents.

Figure S16. Emission spectra of bis-(*p*-H-calix-triprop)-3,6-CBZ (**4**) and bis-(*p*-H-calix-triprop)-2,7-CBZ (**5**) upon continuous irradiation.

Figure S17. UV-Vis spectra of fullerenes C_{60} and C_{70}.

Figure S18. Job plot of complex formation between bis-(*p*-H-calix-triprop)-2,7-CBZ (**5**) and C_{60}/C_{70}.

Figure S19. VT-NMR of bis-(*p*-H-calix-triprop)-3,6-CBZ (**4**) at various temperatures.

Figure S20. VT-NMR of equimolar amounts of bis-(*p*-H-calix-triprop)-3,6-CBZ (**4**) and fullerene C_{60}.

Figure S21. VT-NMR of equimolar amounts of bis-(*p*-H-calix-triprop)-3,6-CBZ (**4**) and fullerene C_{70}.

Figure S22. Stacked ^{1}H NMR spectra of **4**, C_{60}@**4**, and C_{70}@**4** at -10 °C.

Synthesis and Structural Characterization Data of Calixarene-mono-iodo Derivative

Calix[4]arene-tripropyl-mono-iodo derivative **1** was synthesized via selective mono-iodination of 25-hydroxy-26,27,28-tripropoxycalix[4]arene [61] by an adapted synthetic procedure [38]. *p-tert*-Butyl-calix[4]arene [62] and *p*-H-calix[4]arene [63] were obtained according to reported methods.

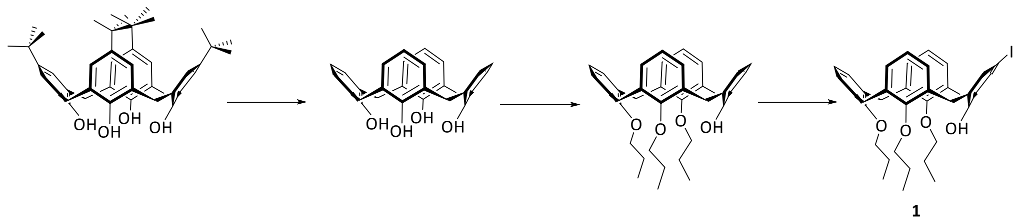

Scheme S1. Synthesis of calix[4]arene-tripropyl-mono-iodo derivative **1**.

Compound **1:** To a solution of 25-hydroxy-26,27,28-tripropoxycalix[4]arene (950 mg, 1.725 mmol) in dry CH_3CN (27 mL) at rt, *p*-toluenesulfonic acid (297.1 mg, 1.725 mmol) was added and allowed to react during 5 min. After that period, *N*-iodosuccinimide (427 mg (1.90 mmol) was introduced in the flask and the resulting yellow suspension was stirring under argon for 17 h at rt, having the TLC control ($CHCl_3$:Hexane (1:1)) revealed the end of the reaction after that period. The solvent was removed in a rotary evaporator and the yellow residue was dissolved in CH_2Cl_2, washed with aqueous solution of $NaHCO_3$ 10% and water and dried. After solvent removal, the crude product was recrystalised from CH_2Cl_2:MeOH, affording 818.2 mg (70%) of **1** as a white solid. *m.p.*: 187-190 °C (*m.p.* lit.[64]: 172.0-172.5 °C); ν_{max}/cm^{-1} (KBr) 3530, 3060, 2963, 2921, 2871, 1588, 1459, 1385, 1292, 1248, 1200, 1158, 1085, 1043, 1005, 963, 908, 845, 799, 762, 626, 592, 554; δ_H ($CDCl_3$, 400.130 MHz) 0.92 (t, 3H, -*O*-CH_2-CH_2-C$\underline{H}_3$, *J* = 7.5 Hz), 1.11 (t, 6H, -*O*-CH_2-CH_2-C$\underline{H}_3$, *J* = 7.4 Hz), 1.82-1.96 (m, 4H, -*O*-CH_2-C$\underline{H}_2$-CH_3), 2.19-2.29 (m, 2H, -*O*-CH_2-C$\underline{H}_2$-CH_3), 3.21 (4H, d, ArC$\underline{H}_{2eq}$Ar, *J*=13.2 Hz), 3.23 (4H, d, ArC$\underline{H}_{2eq}$Ar, *J*=13.6 Hz), 3.72 (4H, t, -*O*-C$\underline{H}_2$-CH_2-CH_3, *J* = 6.7 Hz), 3.82 (t, 2H, -*O*-C$\underline{H}_2$-CH_2-CH_3, *J*=8.4 Hz), 4.30 (d, 4H, ArC$\underline{H}_{2ax}$Ar, *J*=13.8 Hz), 4.39 (d, 4H, ArC$\underline{H}_{2ax}$Ar, *J*=13.1 Hz), 4.86 (s, 1H, ArO$\underline{H}$), 6.31-6.49 (m, 6H, Ar$\underline{H}$), 6.98 (t, 1H, Ar$\underline{H}$, *J* = 7.4 Hz), 7.17 (d, 2H, Ar$\underline{H}$, *J* = 7.4 Hz), 7.40 (s, 2H, Ar$\underline{H}$).

Structural Characterization Data of Calixarene-Carbazole Conjugates

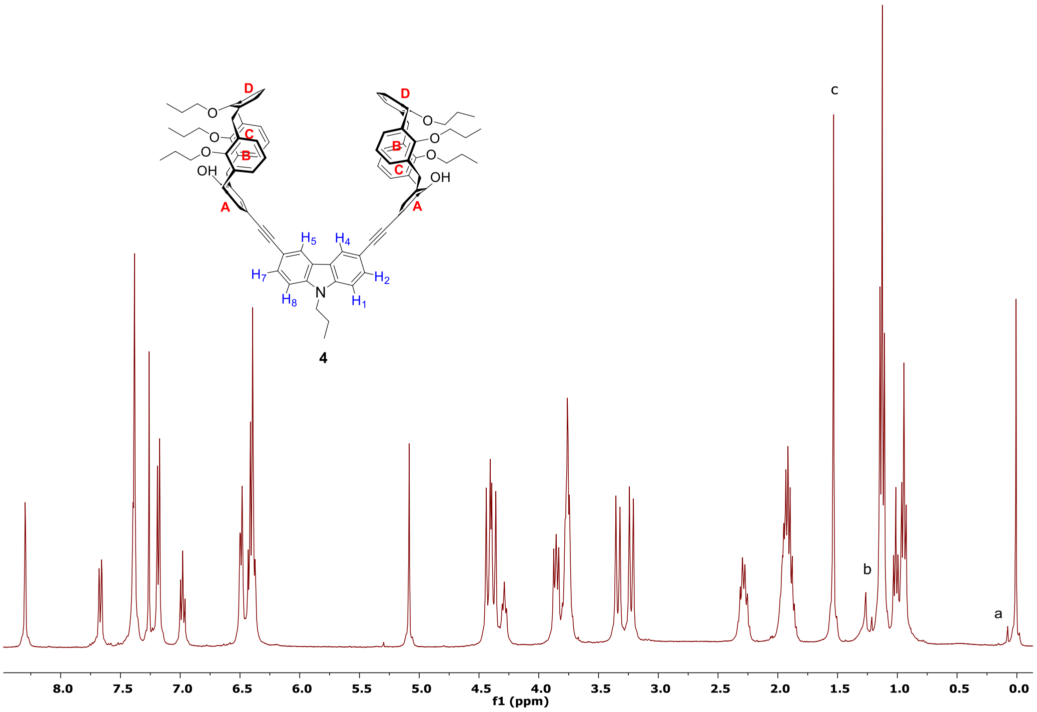

Figure S1. ^{1}H NMR spectrum of bis-(*p*-H-calix-triprop)-3,6-CBZ (**4**) in $CDCl_3$ (400 MHz, 25 ºC) [65]; [a]silicone grease, [b]apiezon type grease, [c]water.

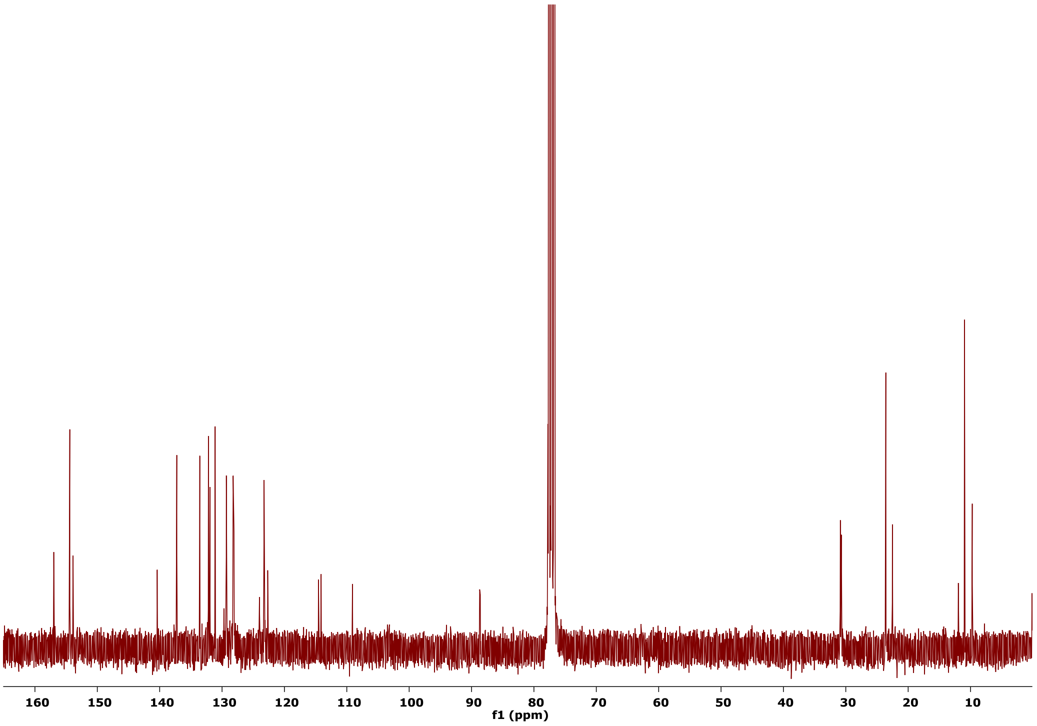

Figure S2. ^{13}C NMR spectrum of bis-(*p*-H-calix-triprop)-3,6-CBZ (**4**) in $CDCl_3$ (75 MHz, 25 ºC) [65].

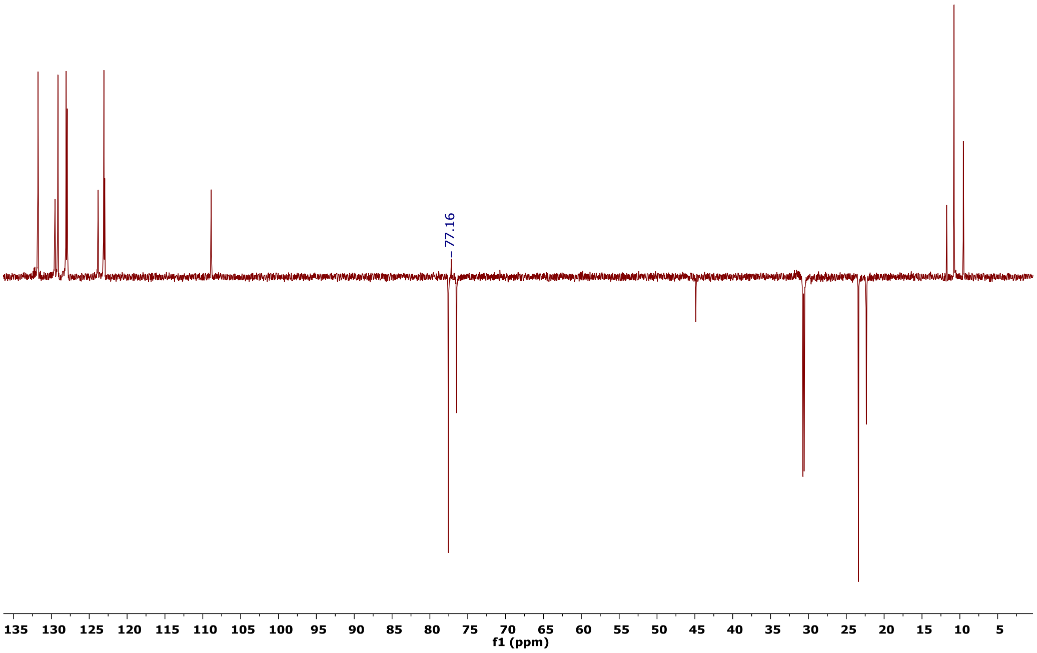

Figure S3. ^{13}C DEPT 135 NMR spectrum of bis-(*p*-H-calix-triprop)-3,6-CBZ (**4**) in $CDCl_3$ (75 MHz, 25 °C) [65].

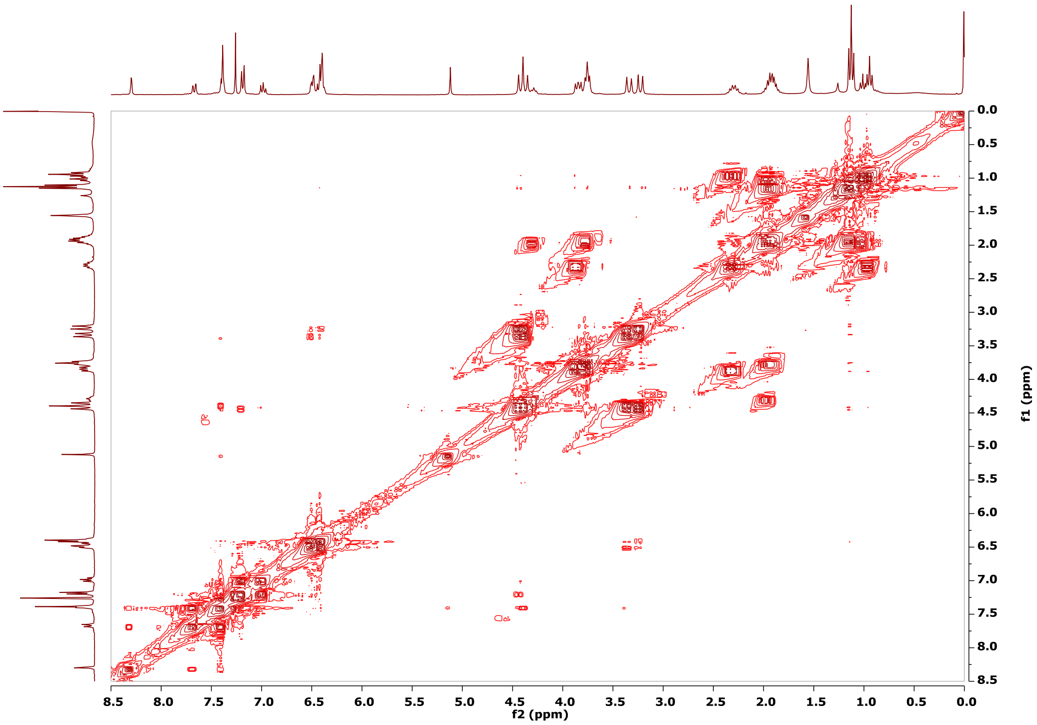

Figure S4. ^1H-^1H COSY NMR spectrum of bis-(*p*-H-calix-triprop)-3,6-CBZ (**4**) in $CDCl_3$ (400 MHz, 25 °C) [65].

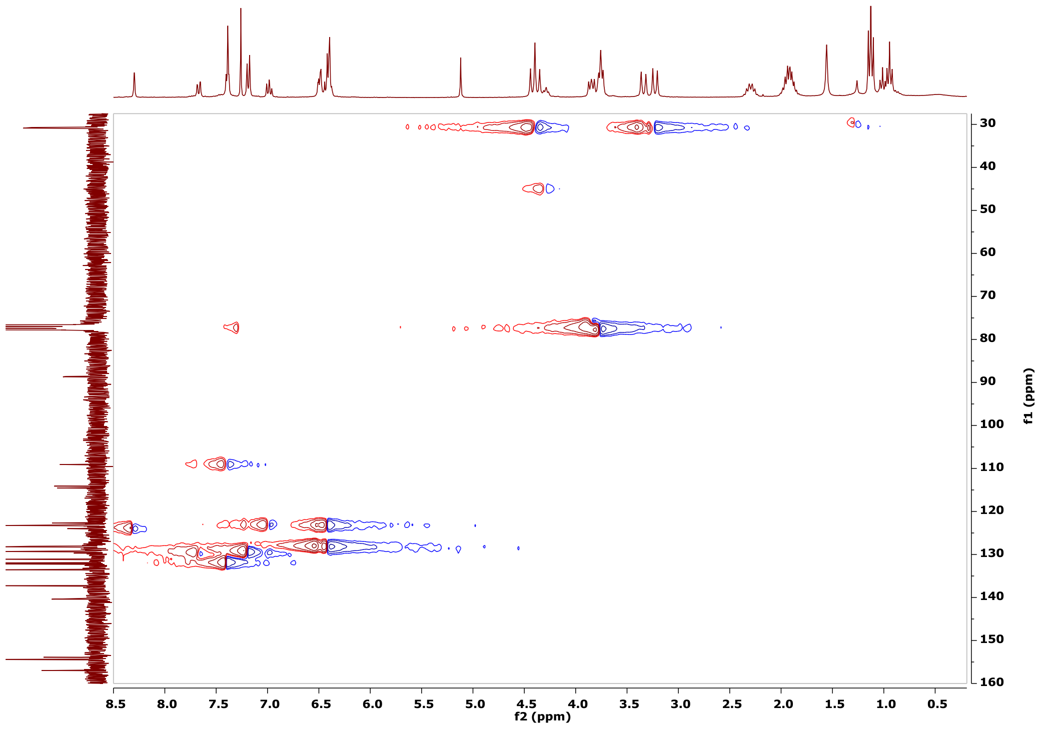

Figure S5. ^{1}H-^{13}C HSQC NMR spectrum of bis-(*p*-H-calix-triprop)-3,6-CBZ (**4**) in $CDCl_3$ (400/75 MHz, 25 ºC) [65].

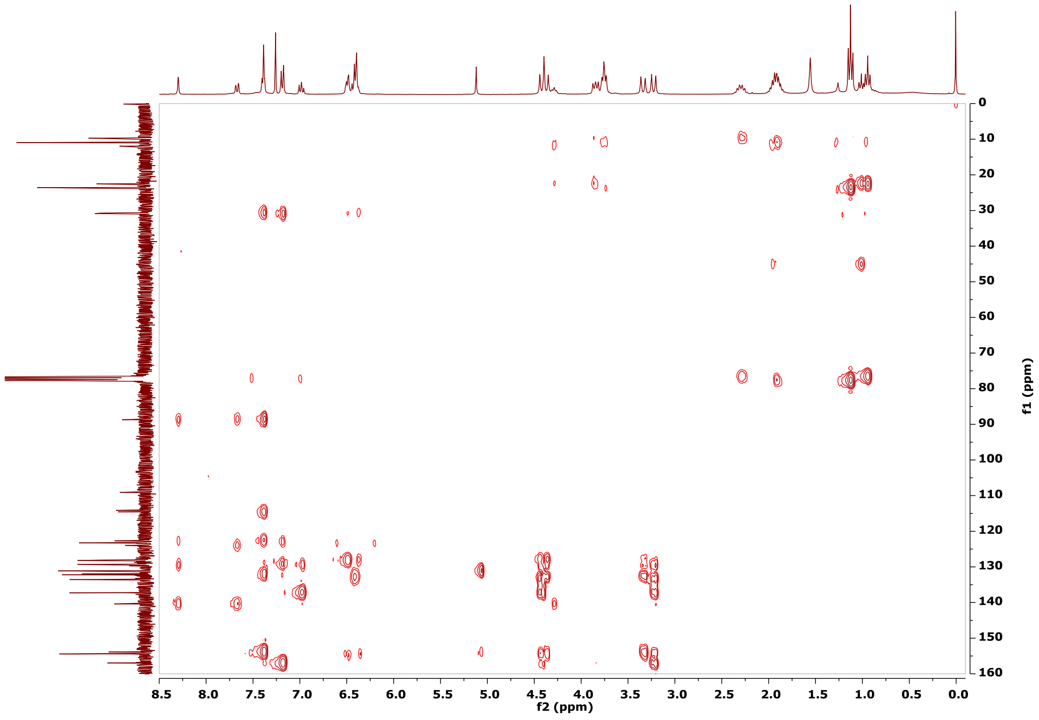

Figure S6. ^{1}H-^{13}C HMBC NMR spectrum of bis-(*p*-H-calix-triprop)-3,6-CBZ (**4**) in $CDCl_3$ (400/75 MHz, 25 ºC) [65].

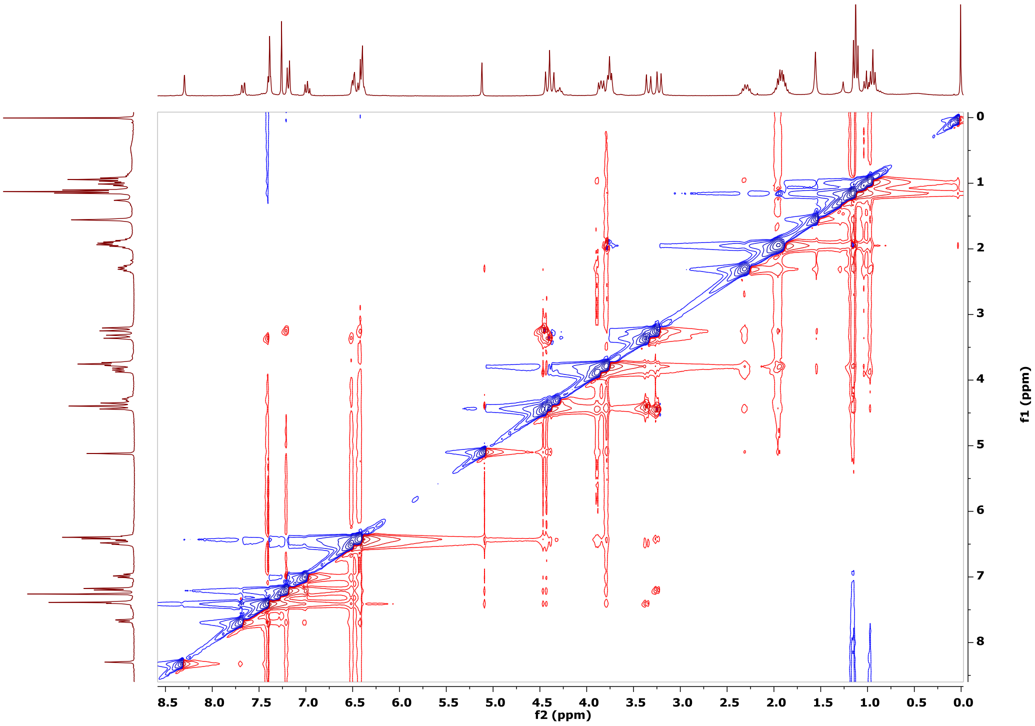

Figure S7. NOESY NMR spectrum of bis-(*p*-H-calix-triprop)-3,6-CBZ (**4**) in $CDCl_3$ (400 MHz, 25 °C) [65].

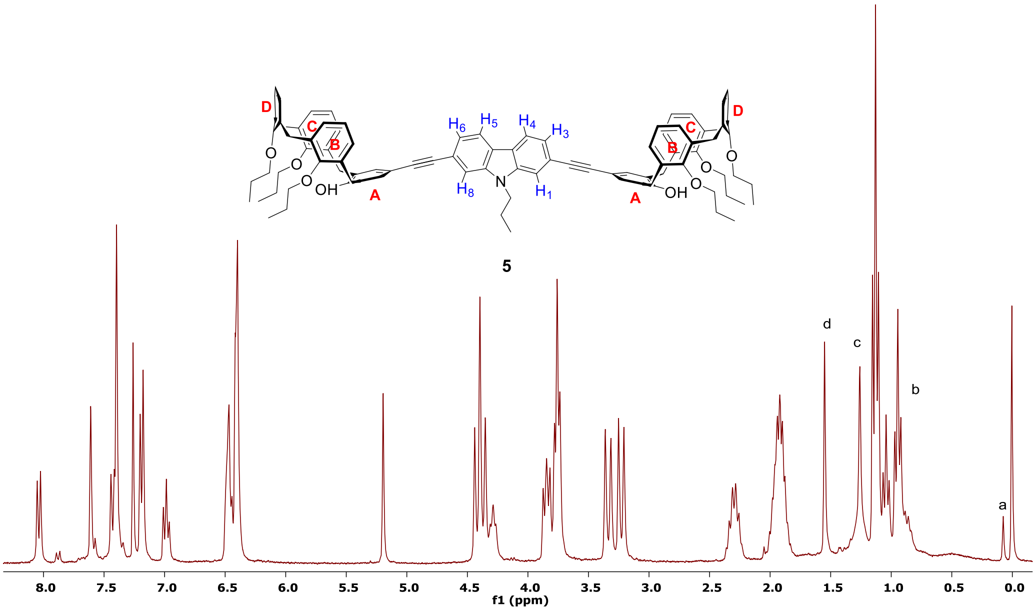

Figure S8. ^1H NMR spectrum of bis-(*p*-H-calix-triprop)-2,7-CBZ (**5**) in $CDCl_3$ (400 MHz, 25 °C) [65]; [a]silicone grease, [b,c]apiezon type grease, [d]water.

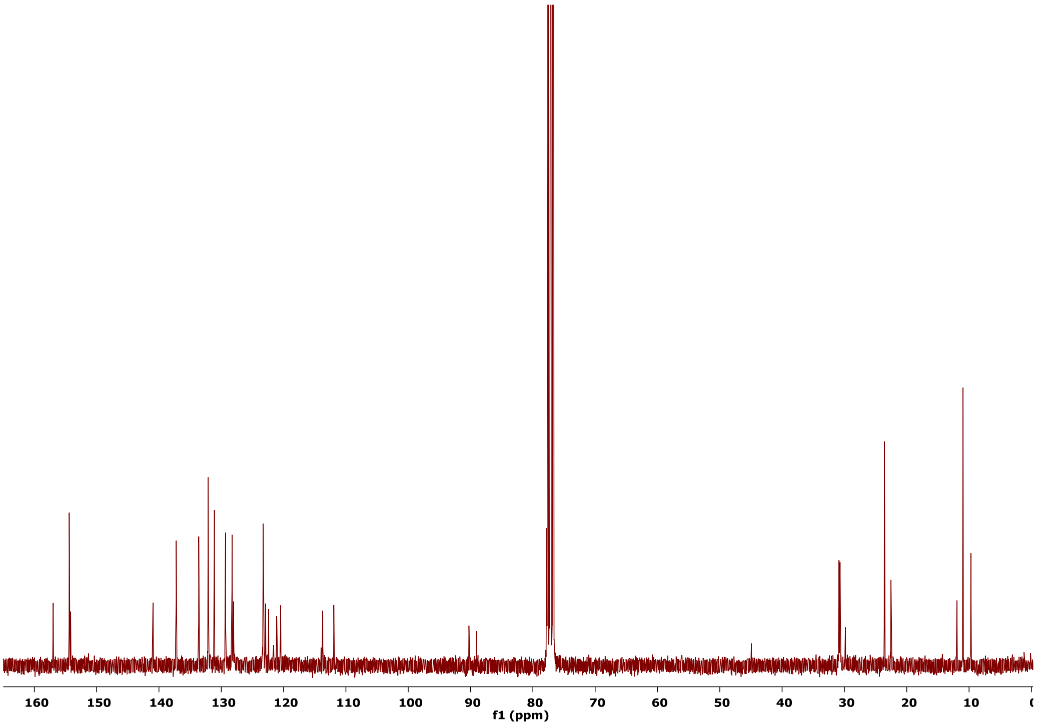

(**a**)

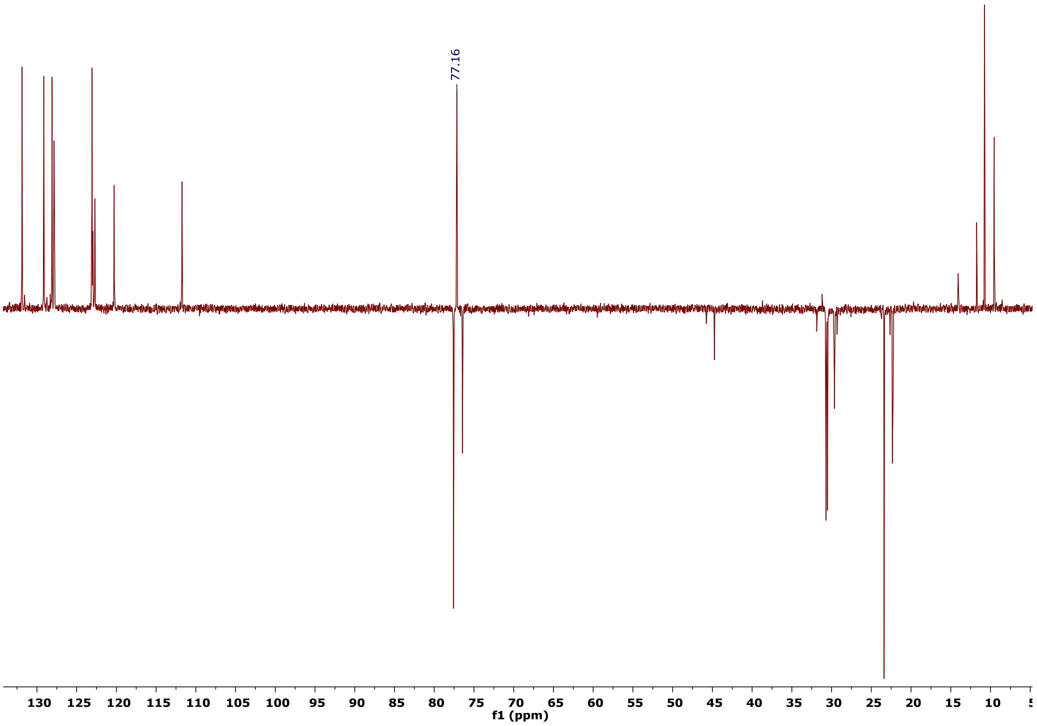

(**b**)

Figure S9. ^{13}C NMR (a) and ^{13}C DEPT 135 NMR (b) spectra of bis-(*p*-H-calix-triprop)-2,7-CBZ (**5**) in $CDCl_3$ (75 MHz, 25 ºC) [65].

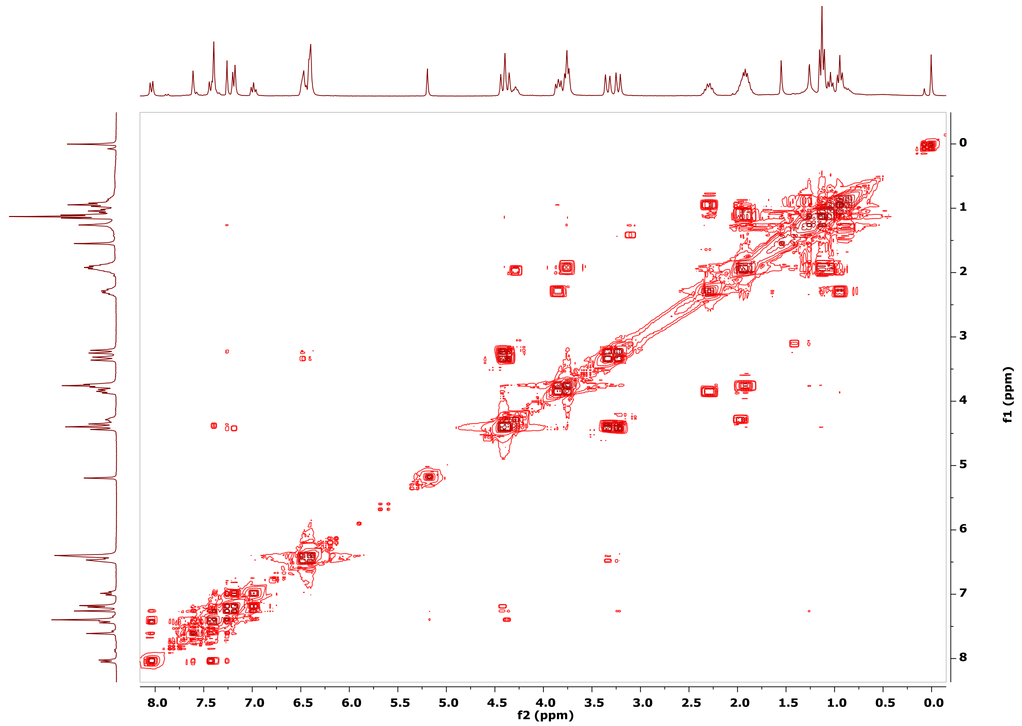

Figure S10. ^{1}H-^{1}H COSY NMR spectrum of bis-(*p*-H-calix-triprop)-2,7-CBZ (**5**) in $CDCl_3$ (300 MHz, 25 ºC) [65].

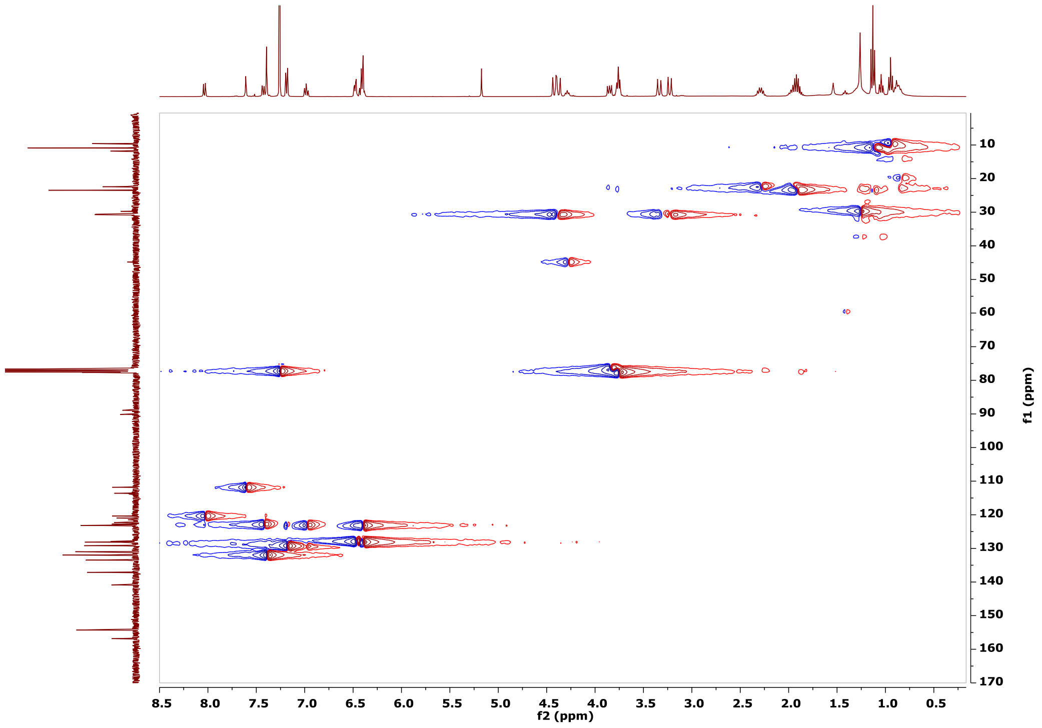

Figure S11. ^{1}H-^{13}C HSQC NMR spectrum of bis-(*p*-H-calix-triprop)-2,7-CBZ (**5**) in $CDCl_3$ (300/75 MHz, 25 ºC) [65].

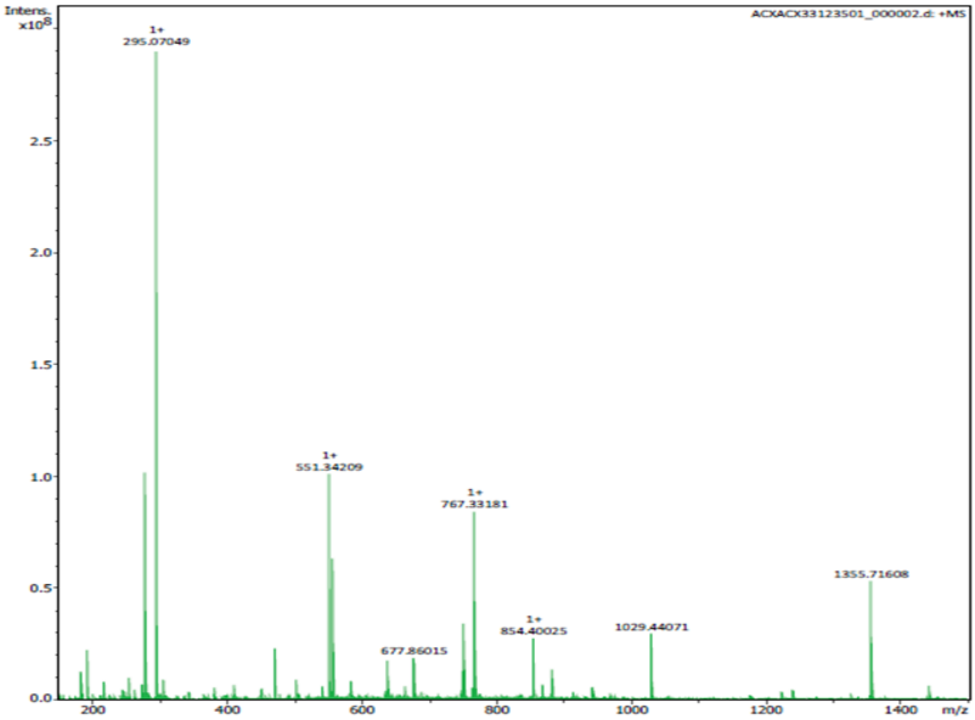

(**a**)

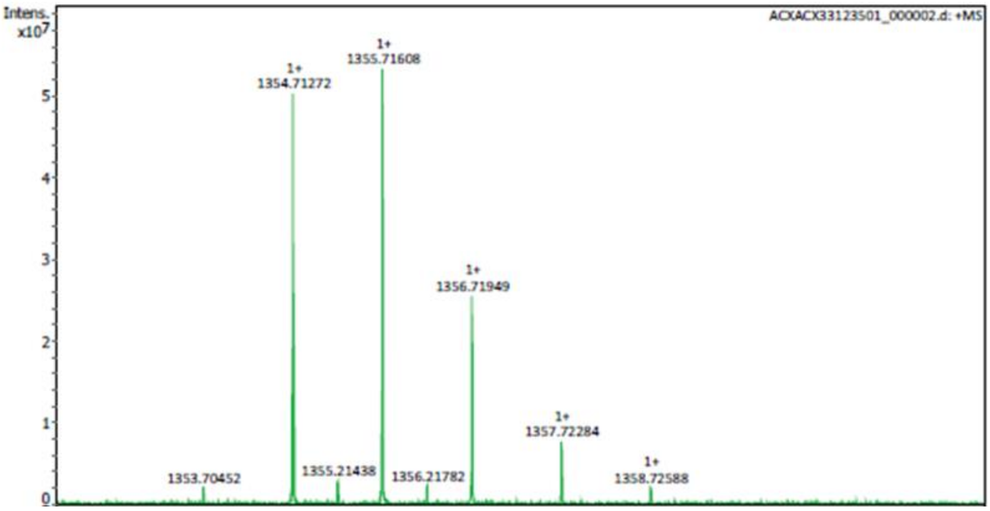

(**b**)

Figure S12. Full ESI-HRMS spectrum of bis-(*p*-H-calix-triprop)-3,6-CBZ (**4**) (**a**); ESI-HRMS spectrum of the molecular ion region (**b**).

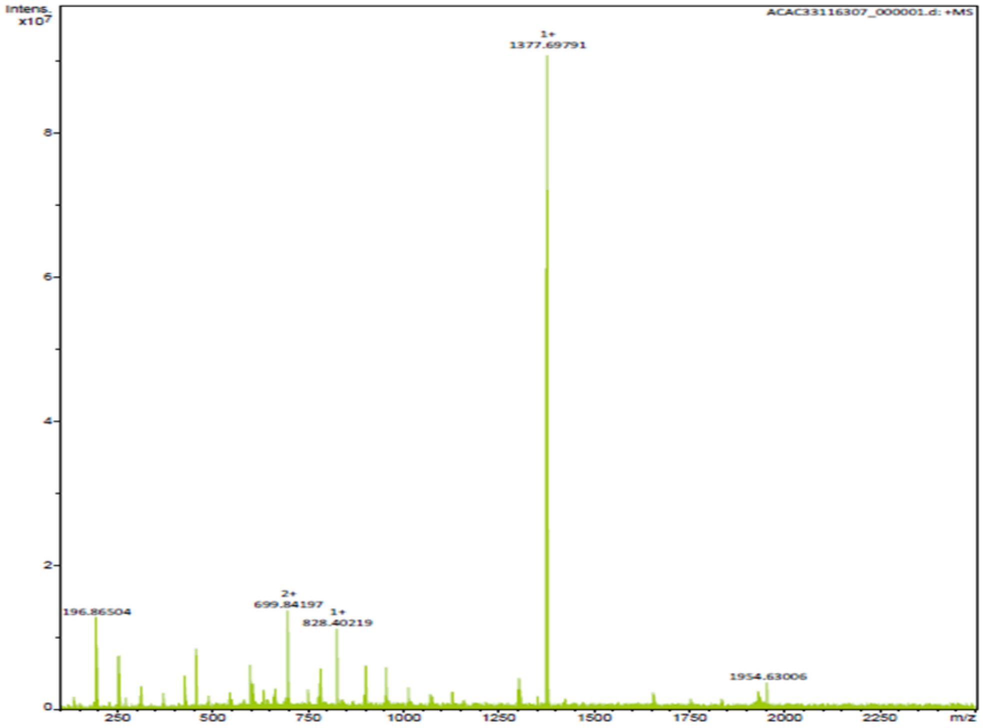

(**a**)

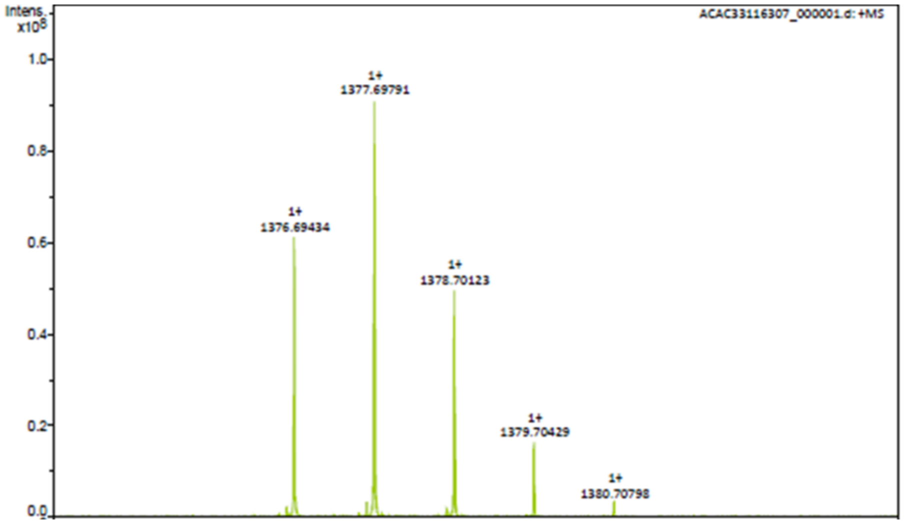

(**b**)

Figure S13. Full ESI-HRMS spectrum of bis-(*p*-H-calix-triprop)-2,7-CBZ (**5**) (**a**); ESI-HRMS spectrum of the molecular ion region (**b**).

Photophysical Properties

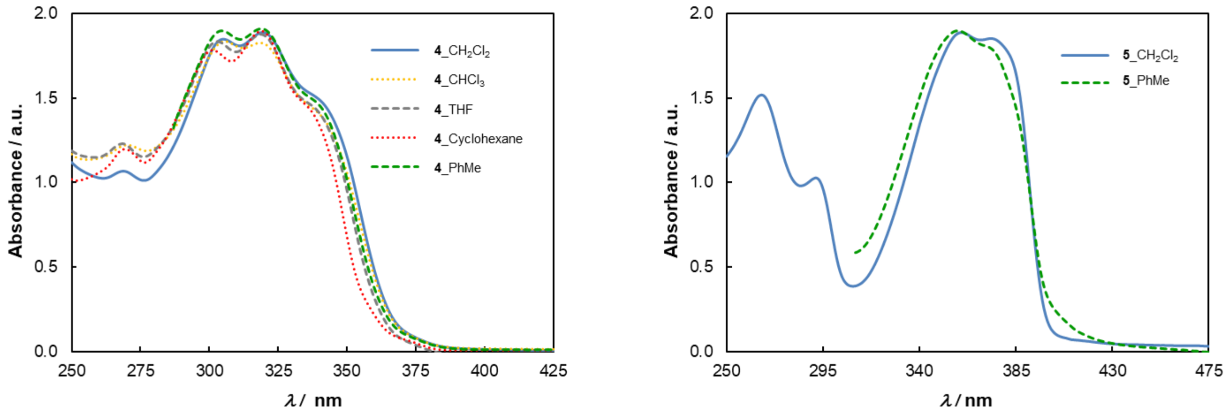

(**a**) (**b**)

Figure S14. UV-Vis spectra of bis-(*p*-H-calix-triprop)-3,6-CBZ (**4**) (**a**) and bis-(*p*-H-calix-triprop)-2,7-CBZ (**5**) (**b**) in different solvents (2.5×10^{-5} M).

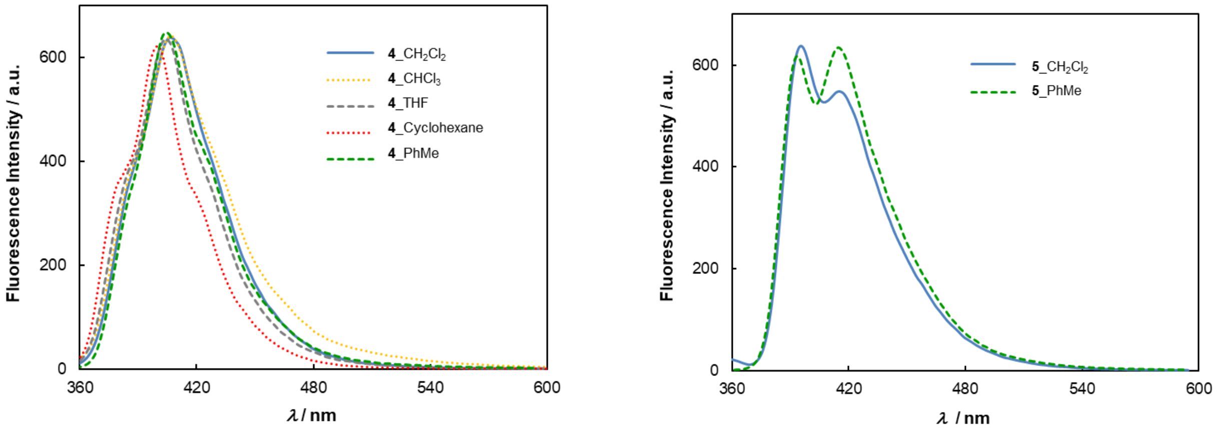

(**a**) (**b**)

Figure S15. Emission spectra of bis-(*p*-H-calix-triprop)-3,6-CBZ (**4**) (**a**) and bis-(*p*-H-calix-triprop)-2,7-CBZ (**5**) (**b**) in different solvents (6.0×10^{-7} M).

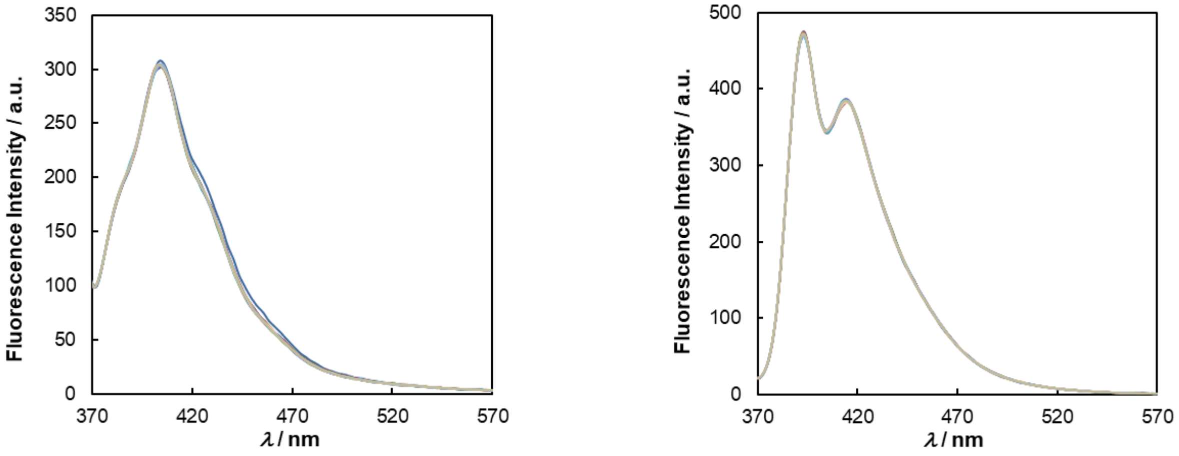

(**a**) (**b**)

Figure S16. Emission spectra of bis-(*p*-H-calix-triprop)-3,6-CBZ (**4**) (**a**) and bis-(*p*-H-calix-triprop)-2,7-CBZ (**5**) (**b**), both in 6.0 × 10^{-7} M in toluene, upon continuous irradiation for for 20 min, under the same experimental conditions carried out on titration experiments (λ_{exc} = 357 nm).

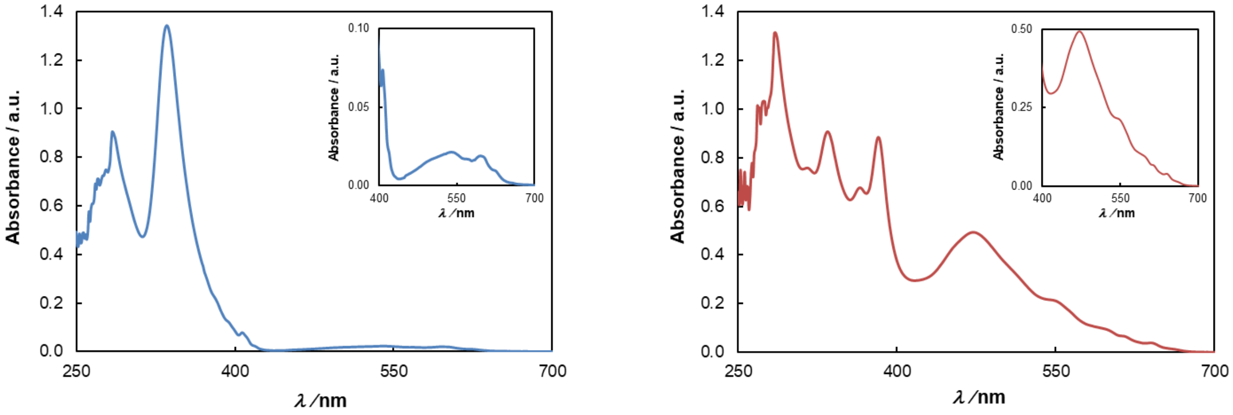

(**a**) (**b**)

Figure S17. UV-Vis spectra of fullerenes C_{60} (**a**) and C_{70} (**b**) in toluene (2.21 x 10^{-5} M); insets: amplification of the region between 400-700 nm.

Job plots

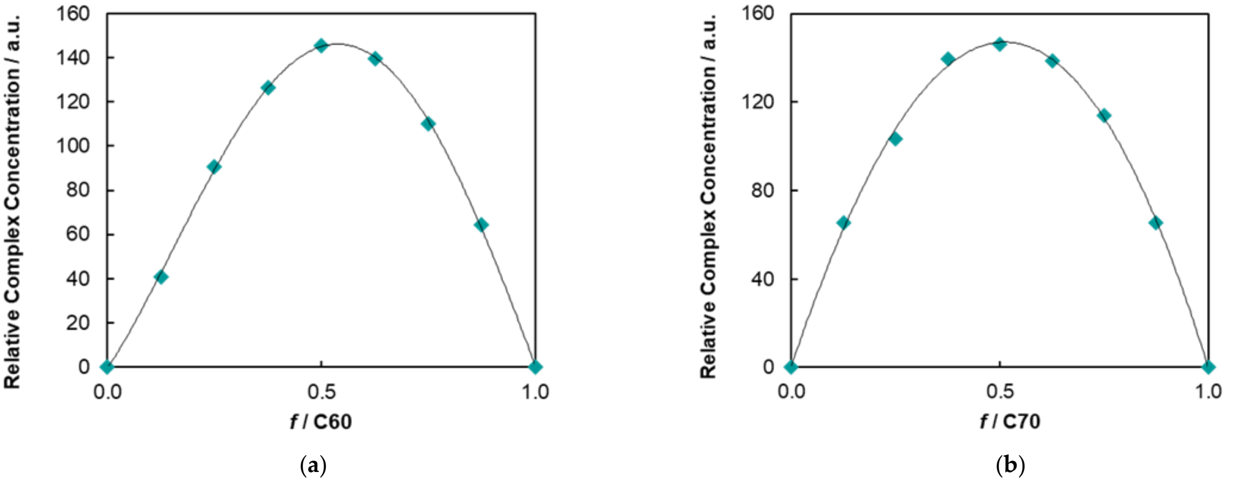

Figure S18. Job plot of complex formation between bis-(*p*-H-calix-triprop)-2,7-CBZ (**5**) and C_{60} (**a**)/C_{70} (**b**) in toluene (at constant 6.0 x 10^{-7} M total concentration) as obtained from changes in fluorescence (λ_{exc} = 357 nm).

Variable-Temperature ^{1}H NMR spectra

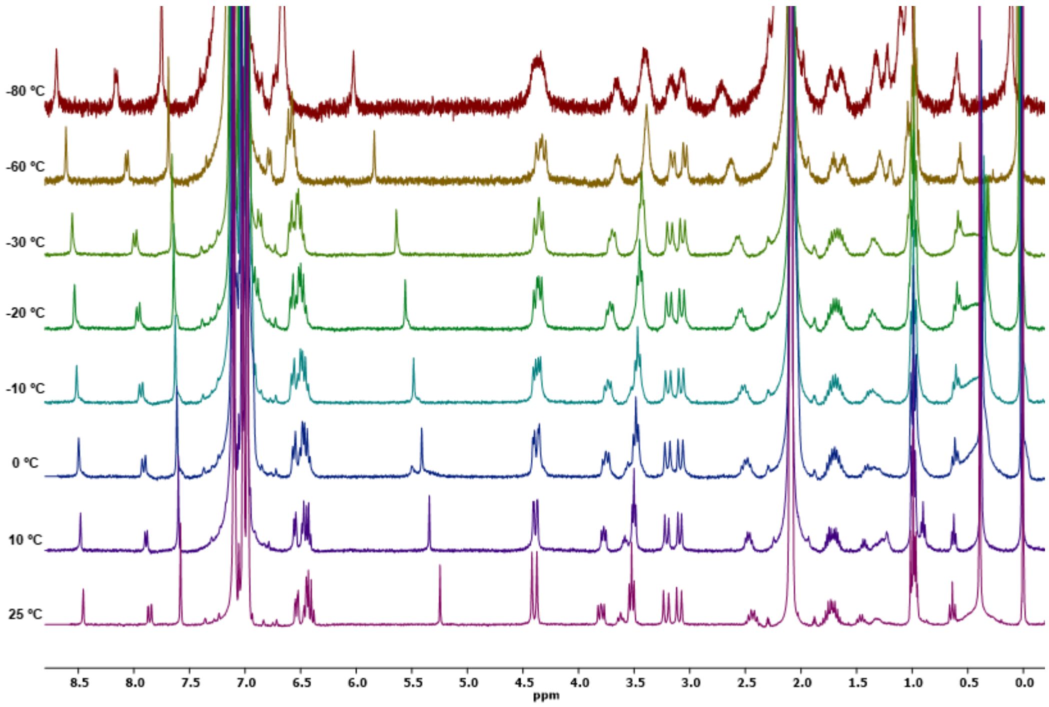

Figure S19. VT-NMR of bis-(*p*-H-calix-triprop)-3,6-CBZ (**4**) at various temperatures (400 MHz, toluene-d_8) [65].

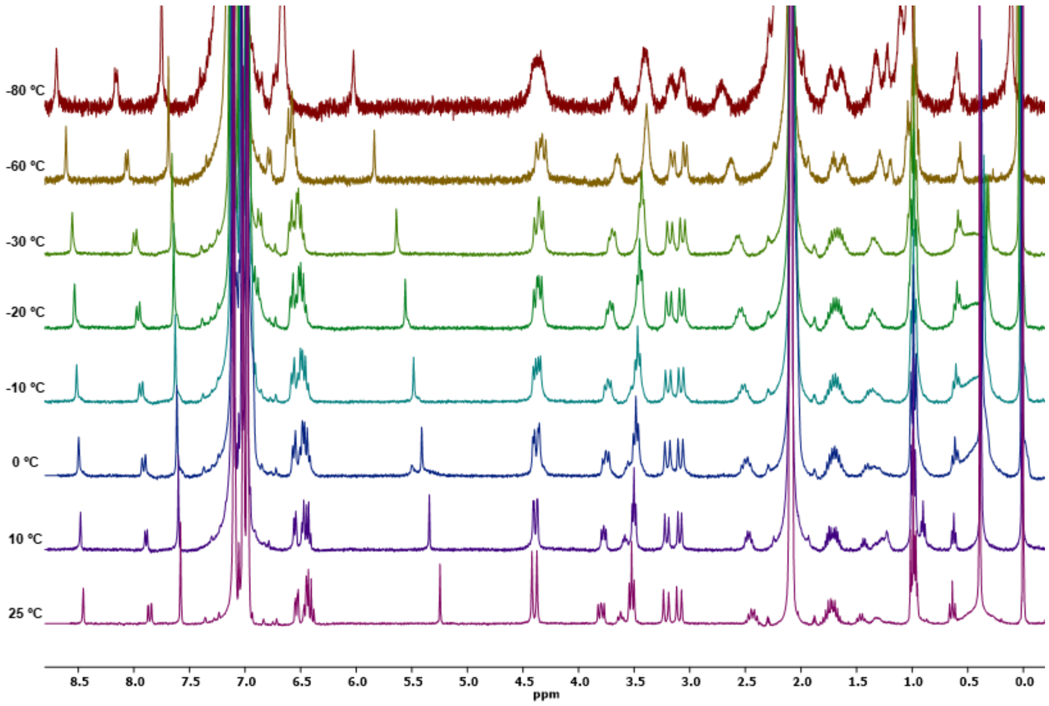

Figure S20. VT-NMR of equimolar amounts of bis-(*p*-H-calix-triprop)-3,6-CBZ (**4**) and fullerene C_{60} at various temperatures (400 MHz, toluene-d_8) [65].

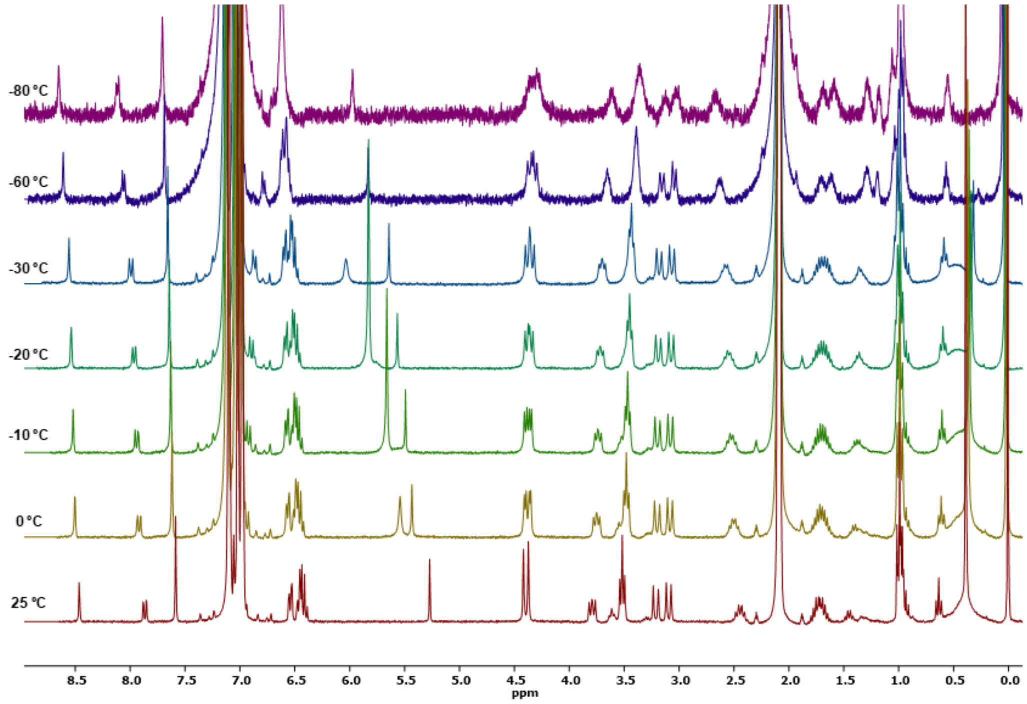

Figure S21. VT-NMR of equimolar amounts of bis-(*p*-H-calix-triprop)-3,6-CBZ (**4**) and fullerene C_{70} at various temperatures (400 MHz, toluene-d_8) [65].

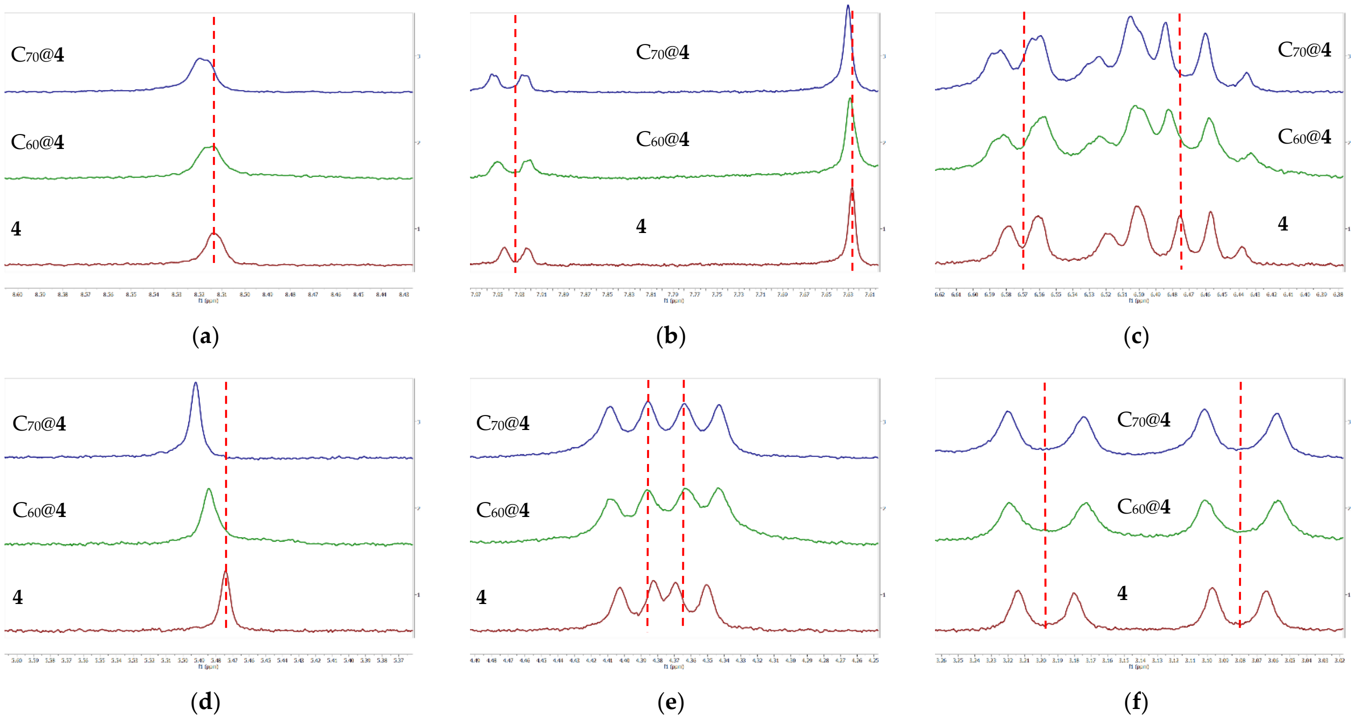

Figure S22. Stacked ^{1}H NMR spectra of **4**, C_{60}@**4**, and C_{70}@**4** at -10 °C, showing complexation-induced shifts (CIS) at several representative regions (400 MHz, toluene-d_8): (**a**) $Ar_{CBZ}\underline{H}_{(4,5)}$; (**b**) $Ar_{CBZ}\underline{H}_{(2,7)}$ and $Ar_{Calix}\underline{H}_{(ring\ A)}$; (**c**) $Ar_{Calix}\underline{H}_{(rings\ B,\ C)}$; (**d**) ArO$\underline{H}$; (**e**) ArC$\underline{H}_{2(ax)}$Ar; (**f**) ArC$\underline{H}_{2(eq)}$Ar [65].

References

38. Bovonsombat, P.; Leykajarakul, J.; Khan, C.; Pla-on, K.; Krause, M. M.; Khanthapura, P.; Ali, R.; Doowa, N. Regioslective iodination of phenol and analogues using *N*-iodosuccinimide and *p*-toluenesulfonic acid. *Tetrahedron Lett*. **2009**, *50*, 2664–2667. https://doi.org/10.1016/j.tetlet.2009.03.128
61. Dondoni, A.; Ghiglione, C.; Marra, A.; Scoponi, M. Synthesis of calix[4]arenylvinylene and calix[4]arenylphenylene oligomers by Stille and Suzuki cross-coupling reactions. *J. Org. Chem*. **1998**, *63*, 9535–9539. https://doi.org/10.1021/jo980868w
62. Gutsche, C.D.; Iqbal, M. *para-tert*-Butylcalix[4]arene. *Org. Synth*. **1990**, *68*, 234–237. https://doi.org/10.15227/orgsyn.068.0234
63. Gutsche, C.D.; Lin, L.G. The Synthesis of Functionalized Calixarenes. *Tetrahedron* **1986**, *42*, 1633–1640. https://doi.org/10.1016/S0040-4020(01)87580-3
64. Gunji, A.; Takahashi, K. Selective and Efficient Iodination of the *p*-Positions of Calix[4]arene Derivatives. *Synth. Commun.* **1998**, *28*, 3933–3941. https://doi.org/10.1080/00397919808004951
65. All NMR spectra were post-processed by MestReNova, version: 14.1.2-25024; Mestrelab Research, S.L., 2020
